# Epigenetic Immune Remodeling of Mesothelioma Cells: A New Strategy to Improve the Efficacy of Immunotherapy

**DOI:** 10.3390/epigenomes5040027

**Published:** 2021-12-14

**Authors:** Maria Fortunata Lofiego, Sara Cannito, Carolina Fazio, Francesca Piazzini, Ornella Cutaia, Laura Solmonese, Francesco Marzani, Carla Chiarucci, Anna Maria Di Giacomo, Luana Calabrò, Sandra Coral, Michele Maio, Alessia Covre, on behalf of the EPigenetic Immune-Oncology Consortium Airc (EPICA) Investigators

**Affiliations:** 1 Center for Immuno-Oncology, Medical Oncology and Immunotherapy, Department of Oncology, University Hospital of Siena, 53100 Siena, Italy; mariafl@hotmail.it (M.F.L.); saracannito@hotmail.it (S.C.); carolinafazio137@gmail.com (C.F.); f.piazzini.92@gmail.com (F.P.); cutaiaornella@gmail.com (O.C.); francesco.marzani92@gmail.com (F.M.); carlabio@hotmail.it (C.C.); a.m.digiacomo@ao-siena.toscana.it (A.M.D.G.); l.calabro@ao-siena.toscana.it (L.C.); scoral2@yahoo.it (S.C.); mmaiocro@gmail.com (M.M.); 2 Epigen Therapeutics S.R.L., 53100 Siena, Italy; solmonese.laura@gmail.com; 3 Medical Oncology, Department of Molecular and Developmental Medicine, University of Siena, 53100 Siena, Italy

**Keywords:** epigenetic drugs, DNA methylation, immunotherapy, malignant pleural mesothelioma

## Abstract

Malignant pleural mesothelioma (MPM) is an aggressive malignancy with a severe prognosis, and with a long-standing need for more effective therapeutic approaches. However, treatment with immune checkpoint inhibitors is becoming an increasingly effective strategy for MPM patients. In this scenario, epigenetic modifications may negatively regulate the interplay between immune and malignant cells within the tumor microenvironment, thus contributing to the highly immunosuppressive contexture of MPM that may limit the efficacy of immunotherapy. Aiming to further improve prospectively the clinical efficacy of immunotherapeutic approaches in MPM, we investigated the immunomodulatory potential of different classes of epigenetic drugs (i.e., DNA hypomethylating agent (DHA) guadecitabine, histone deacetylase inhibitors VPA and SAHA, or EZH2 inhibitors EPZ-6438) in epithelioid, biphasic, and sarcomatoid MPM cell lines, by cytofluorimetric and real-time PCR analyses. We also characterized the effects of the DHA, guadecitabine, on the gene expression profiles (GEP) of the investigated MPM cell lines by the nCounter platform. Among investigated drugs, exposure of MPM cells to guadecitabine, either alone or in combination with VPA, SAHA and EPZ-6438 demonstrated to be the main driver of the induction/upregulation of immune molecules functionally crucial in host-tumor interaction (i.e., HLA class I, ICAM-1 and cancer testis antigens) in all three MPM subtypes investigated. Additionally, GEP demonstrated that treatment with guadecitabine led to the activation of genes involved in several immune-related functional classes mainly in the sarcomatoid subtype. Furthermore, among investigated MPM subtypes, DHA-induced CDH1 expression that contributes to restoring the epithelial phenotype was highest in sarcomatoid cells. Altogether, our results contribute to providing the rationale to develop new epigenetically-based immunotherapeutic approaches for MPM patients, potentially tailored to the specific histologic subtypes.

## 1. Introduction

Malignant pleural mesothelioma (MPM) is a low-frequency thoracic neoplasm arising from mesothelial cells of the pleural cavity, featuring a high aggressiveness (5-year survival rate of about 5%) [1]. The prognosis is poor, with an almost invariably fatal course within 24 months from the diagnosis and few long-term survivors [2]. MPM exhibits the histological subdivision of MPM into epithelioid, sarcomatoid and biphasic subtypes comprising about 50–60%, 10–20% and 25–35% of all MPM, respectively [3]. The histopathology strongly influences patients’ survival, thus defining a crucial prognostic factor. The best outcome has been observed for the epithelioid variant; conversely, the sarcomatoid phenotype portends a particularly dismal prognosis; finally, the biphasic subtype shows an intermediate survival, depending on the prevalent component of the tumor [4,5]. This histological differentiation of MPM cells suggests that the epithelial-to-mesenchymal transition (EMT) process, by which epithelial cells lose their polarity and cell contacts, acquire the expression of mesenchymal markers, and manifest a migratory phenotype, represents an event in MPM progression [6,7]. Moreover, a substantial switch from epithelial markers (E-cadherin) to mesenchymal markers (N-cadherin) through epithelioid to biphasic and sarcomatoid subtypes was demonstrated, suggesting the potential usefulness of these EMT-markers in the diagnosis of mesothelioma [8,9].

Despite the accumulation of novel insights about MPM biology [10,11] and the considerable number of ongoing clinical investigations [12], MPM is still an aggressive cancer with a dismal prognosis and limited clinical benefits. Immune check-point inhibitors (ICI) therapy represents an attractive strategy in the treatment of solid tumors in particular when given in combination regimes. The clinical efficacy of combined ICI therapies in MPM patients was firstly proved in the phase II NIBIT-MESO-1 study, investigating the combination of the anti-CTLA-4 monoclonal antibody (mAb) tremelimumab with the anti-PD-L1 mAb durvalumab, as first-/second-line therapy, registering a median overall survival (OS) of 16.6 months [13]. These results were then confirmed by two other combination studies: the phase II MAPS-2 trial, comparing the anti-CTLA-4 mAb ipilimumab and the anti-PD-1 mAb nivolumab vs. nivolumab alone [14]; the single-arm phase II INITIATE study, investigating the combination of ipilimumab plus nivolumab in MPM patients with disease progression or recurrence after platinum-based treatment [15]. Further support to the efficacy of combined ICI therapy in MPM derived from the results of the phase III CheckMate 743 trial, in which first-line nivolumab plus ipilimumab significantly improved the survival of patients when compared to standard chemotherapy [16]. Accordingly, the latest report of the NIBIT-MESO-1 study revealed the potential efficacy and safety of retreatment with tremelimumab and durvalumab in MPM patients who experienced disease progression after an initial clinical benefit (i.e., disease control) [17]. Despite these promising results, a proportion of patients still do not respond to ICI therapy, possibly due to multiple mechanisms of primary and secondary resistance to the treatment [18]. These could include epigenetic derangements that are known to contribute to the pathogenesis of MPM and to its highly immunosuppressive microenvironment [19,20,21].

Epigenetic modifications include principally DNA methylation, post-translational modifications (PTMs) of histone proteins, chromatin remodeling components, histone variant exchange, and non-coding RNAs [5,22]. Unlike genetic mutations, epigenetic alterations are potentially reversible and have great plasticity, thus the possibility of changing tumor immune contexture through epigenetic compounds represents a promising strategy to improve the efficacy of cancer immunotherapy. Along this line, it has been highly demonstrated that epigenetic remodeling of cancer cells by DNA hypomethylating agents (DHA), in particular decitabine and guadecitabine, induced/up-regulated the expression of different immune molecules (i.e., HLA class I, cancer testis antigens (CTA), co-stimulatory molecules, interferon stimulated genes) in cancer cells of different histotypes including MPM [23,24,25], resulting in an improved recognition of tumor cells by immune cells [26,27,28,29,30] that suggested the promising role of DHA to improve the clinical effectiveness of cancer immunotherapies [31]. Additionally, the use of histone deacetylase inhibitors (HDACi) is under clinical and preclinical evaluation for cancer treatment, either as monotherapy or in the combinatorial setting. Specifically, valproic acid (VPA) was demonstrated to be able to decrease the immunosuppressive activity of myeloid-derived suppressor cells, either in vitro or in vivo, in a murine model [32]; the administration of low-dose of trichostatin-A, potentiated the anti-tumor activity of infiltrating macrophages in various tumors, favoring the M1-like phenotype and reshaping the tumor microenvironment (TME) [33]; also, VPA and suberoylanilide hydroxamic acid (SAHA) triggered cell death of epithelioid MPM cells, and synergized with DHA to induce CTA expression in the remaining living cells, which became sensitive to lysis mediated by CTA-specific cytotoxic T-cells [34]. HDAC inhibitors can further synergize with DHA to induce endogenous retrovirus transcription, promote chemokine-dependent T cell infiltration and favor T cell memory and effector phenotypes, thereby reverting immune evasion in non-small-cell lung carcinoma (NSCLC) models [35].

Other epigenetic compounds have been demonstrated to modulate immune cell transcriptional programs. Tazemetostat (EPZ-6438), an inhibitor of the enhancer of zeste homologue 2 (EZH2i) favored chemokine-dependent T cell attraction as a consequence of enhanced expression of the immunostimulatory molecules in cancer [35]. Furthermore, EZH2 was critical for tumor-infiltrating T regulatory lymphocytes (Treg) immunosuppressive functions, and its pharmacologic inhibition not merely reprogrammed Treg activity, but also led to enhanced CD8+T cell response within a murine colorectal tumor [36].

Based on the encouraging sensitivity of MPM to immunotherapy and on the promising immunomodulatory effects of epigenetic remodeling, this study focused to compare the properties of different classes of epigenetic drugs, including agents recently used in clinical trials (i.e., guadecitabine, EPZ-6438), on MPM cell lines belonging to the three main subtypes. The identification of guadecitabine as the main driver of the greatest immunomodulatory effects led us to expand the characterization of the DHA activity by analyzing changes in gene expression profiles (GEP) of MPM cell lines belonging to the three subtypes and elucidating the biologic functions of genes representing the expression signature of epigenetically-treated vs. untreated MPM cells. Results obtained provide the rationale for the development of new more effective epigenetic-based immunotherapeutic strategies for the treatment of MPM patients also relating to their histologic prognosis.

## 2. Results

### 2.1. Analysis of HLA Class I Antigens and ICAM-1 Expression on MPM Cell Lines Treated with Epigenetic Drugs

**Figure 1:**
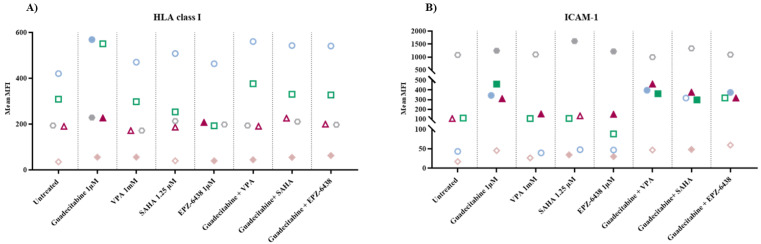
Flow cytometry analysis of changes induced by different epigenetic drugs in HLA class I and ICAM-1 molecules expression. MPM cell lines untreated or treated with guadecitabine, VPA, SAHA, EPZ-6438, or guadecitabine-based combinations were incubated with (**A**) anti-human HLA class I or (**B**) anti-human ICAM-1 mAbs and studied by flow cytometry. Data obtained were analyzed by Kaluza software. Values reported correspond to MFI in treated vs. untreated cells. Each data point represents mean value of MFI obtained in 3 independent experiments for each single cell line.

To explore the immunomodulatory activity of different classes of epigenetic drugs on MPM cell lines, cytofluorimetric analyses of the expression of HLA class I antigens and of the costimulatory molecule ICAM-1 have been conducted on 5 MPM cell lines untreated or treated with selected DHA, HDACi, or EZH2i, alone or in combination (Figure S1). Results obtained showed an up-regulation of HLA class I antigens in all MPM cells treated with guadecitabine that was statistically significant (*p* ≤ 0.05) in 4/5 (two sarcomatoid, 1 biphasic and 1 epitheliod) treated cell lines, as compared to untreated cells (Figure 1A, Table S1). Conversely, only a sporadic up-regulation of HLA class I antigens expression was observed in MPM cell lines treated with other investigated epigenetic drugs, and a statistically significant difference was detected in one sarcomatoid cell line treated with VPA and in two sarcomatoid cell lines treated with EPZ-6438, used alone (Figure 1A, Table S1). Moreover, the combination of different epigenetic drugs with guadecitabine did not enhance the up-regulated expression of HLA class I induced by a single treatment. In fact, a significant difference in HLA class I expression was induced only in one sarcomatoid cell line by guadecitabine plus VPA, SAHA, or EPZ-6438 combination treatments (Figure 1A, Table S1). No differences in the percentage of HLA class I antigens positive cells was observed after any treatment, in all investigated MPM cell lines (data not shown).

Similar to HLA class I antigens, ICAM-1 expression was upregulated in all MPM cell lines treated by guadecitabine alone and this up-regulation was statistically significant (*p* ≤ 0.05) in 4/5 (one sarcomatoid, one biphasic and two epitheliod) treated cell lines, as compared to untreated cells (Figure 1B, Table S1). Additionally, in this case, a minor modulatory effect of ICAM expression was induced by treatment with other investigated epigenetic drugs. In detail, a statistically significant up-regulation was detected in one sarcomatoid cell line treated with VPA and in 2/4 (one sarcomatoid and one biphasic) cell lines treated with SAHA or EPZ-6438, used alone (Figure 1B, Table S1). Again, the combination of different epigenetic drugs with guadecitabine did not enhance the up-regulated expression of ICAM-1 induced by single treatment. In fact, a significant difference in ICAM-1 expression was observed in 3/4 MPM cell lines treated with guadecitabine plus VPA (one sarcomatoid and two epithelioid) or SAHA (two sarcomatoid and one epithelioid) and in 2/4 (one sarcomatoid and one epithelioid) MPM cell lines treated with guadecitabine plus EPZ-6438 (Figure 1B, Table S1).

Lastly, guadecitabine and guadecitabine-based combinations up-regulated the percentage of ICAM-1-positive cells in the sarcomatoid MPM cell line with the lowest percentage of ICAM-1 positive cells and did not affect other investigated MPM cell lines. No differences were induced in the percentage of ICAM-1 positive cells by other epigenetic drugs in all MPM cell lines (Table S1).

### 2.2. Molecular Analysis of CTA Expression in MPM Cell Lines Treated with Epigenetic Drugs

**Figure 2:**
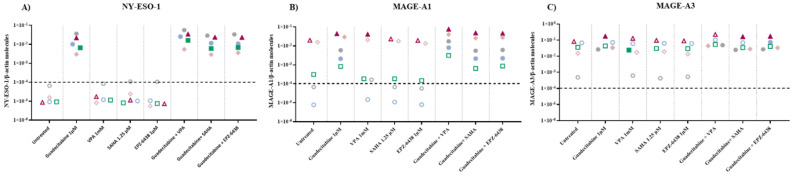
Quantitative real-time PCR analysis of changes induced by different epigenetic drugs in CTA expression. Total RNA was extracted from MPM cell lines, untreated or treated with guadecitabine, VPA, SAHA, EPZ-6438, or guadecitabine-based combinations. Quantitative real-time PCR analyses were performed on retrotranscribed total RNA, utilizing CTA- and β-actin-specific primers. Scatter plots report mean number of molecules of (**A**) NY-ESO-1, (**B**) MAGE-A1, and (**C**) MAGE-A3 in treated and untreated MPM cell lines. Values are reported as specific CTA/β-actin mRNA molecules. Each data point represents mean value of molecules obtained in 3 independent experiments for each single cell line. Dotted line represents the cut-off of positive gene expression value ≥ 10 × 10^−4^. Full shape, *p*-Value ≤ 0.05 (calculated by Student *t*) vs. untreated cells; empty shape, *p*-Value >0.05.

Quantitative real-time RT-PCR analyses were performed to investigate changes in the expression levels of CTA (i.e., NY-ESO-1, MAGE-A1, and MAGE-A3) induced by the different investigated epigenetic drugs alone or in combinations, in 5 MPM cell lines. Baseline levels of NY-ESO-1 and MAGE-A1 were negative in five and two (one epithelioid and one biphasic) cell lines, respectively; all cell lines were constitutively positive for MAGE-A3 expression. A de novo expression of NY-ESO-1 was induced by guadecitabine and guadecitabine-based combinations in 5/5 MPM cell lines (Figure 2A; Table S2). No induction in the expression of NY-ESO-1 gene was observed with HDACi or EZH2i treatment, except for the biphasic Meso4 cell line after SAHA or EPZ-6438 treatments (Figure 2A; Table S2). Guadecitabine and guadecitabine-based combinations induced the expression of MAGE-A1 in the two constitutively antigen-negative cell lines, and significantly (*p* ≤ 0.05) up-regulated the expression in the two constitutively positive sarcomatoid cell lines (Figure 2B; Table S2). No induction nor upregulation in the expression of MAGE-A1 was observed with HDACi or EZH2i treatment, except for the biphasic cell line and for one sarcomatoid cell line, in which MAGE-A1 expression was induced and significantly upregulated, respectively, by VPA (Figure 2B; Table S2). Finally, MAGE-A3 expression was significantly upregulated in three (two sarcomatoid and one biphasic) cell lines by guadecitabine and in two (one sarcomatoid and one biphasic), three (two sarcomatoid and one biphasic) and four (two sarcomatoid, one biphasic and one epithelioid) cell lines by guadecitabine combined with VPA, SAHA and EPZ-6348, respectively (Figure 2B; Table S2). No significant modulation in MAGE-A3 expression was observed with HDACi or EZH2i treatment, except for Meso6 showing a significant decrease in antigen expression after treatment with VPA (Figure 2C; Table S2).

### 2.3. Molecular Analysis of Gene Expression of EMT-Regulating Genes on MPM Cell Lines Treated with Epigenetic Drugs

**Figure 3:**
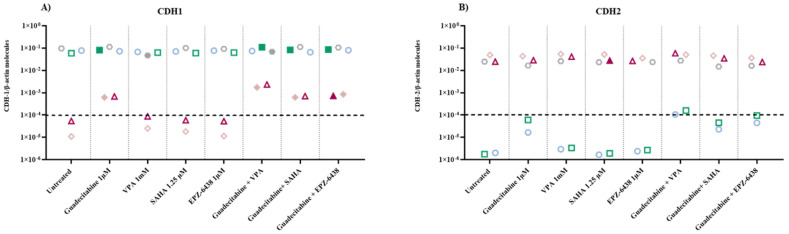
Quantitative real-time PCR analysis of changes induced by different epigenetic drugs in the expression of cadherin-coding genes. Total RNA was extracted from MPM cell lines, untreated or treated with guadecitabine, VPA, SAHA, EPZ-6438, and guadecitabine-based combinations. Quantitative real-time PCR analyses were performed on retrotranscribed total RNA, utilizing CDH1-, CDH2- and β-actin-specific primers. Scatter plots report the mean number of molecules of (**A**) CDH1 and (**B**) CDH2 in treated and untreated MPM cell lines. Values are reported as specific cadherins/β-actin gene. Each data point represents the mean value of molecules obtained in 3 independent experiments for each single cell line. The dotted line represents the cut-off of positive gene expression value ≥10 × 10^−4^. Full shape, *p*-Value ≤0.05 (calculated by Student *t*) vs. untreated cells; empty shape, *p*-Value >0.05.

To study the effect of different epigenetic drugs on the EMT phenomenon, quantitative real-time RT-PCR analyses were performed to investigate changes in the expression levels of E-cadherin/CDH1 and N-cadherin/CDH2, induced by the different investigated epigenetic drugs and their combinations, in five MPM cell lines. Results showed that the constitutive expression of the CDH1 gene was negative in the sarcomatoid cell lines, and highly positive in both biphasic and epithelial MPM cell lines (Figure 3A; Table S2). The expression of CDH1 was induced in the sarcomatoid cell lines only by guadecitabine or guadecitabine-based combinations. A significant (*p* ≤ 0.05) up-regulation of CDH1 expression was observed in 1 (epithelioid)/3 CDH1-positive cell lines after guadecitabine and guadecitabine-combined treatments, and in one biphasic cell line treated with VPA or guadecitabine plus VPA. Other treatments did not induce any modulation of CDH1 expression in investigated cell lines (Figure 3A; Table S2). Conversely, constitutive expression of the CDH2 gene was negative in the epithelial cell lines, and positive in both sarcomatoid and biphasic MPM cell lines (Figure 3B; Table S2). The expression of CDH2 was induced only by guadecitabine plus VPA in the two constitutively antigen-negative epithelioid cells and significantly upregulated only by guadecitabine plus SAHA combination in one sarcomatoid cell line compared to control. Importantly, no induction or up-regulation in CDH2 expression was observed after treatment with guadecitabine alone (Figure 3B; Table S2).

### 2.4. nCounter Gene Expression Panel Analysis

The GEP of 10 MPM cell lines untreated or treated with guadecitabine was evaluated using the NanoString PanCancer IO 360 panel on the nCounter SPRINT Profiler. Results demonstrated that, among 770 investigated genes, a mean of 300.8 (range: 236–375) genes were differentially (Log2 ratio ≥ 0.58; Log2 ratio ≤ −0.58) expressed in treated vs. untreated cells. Mean of 68.3% (range: 46.7–83.7%) and 31.7% (range: 16.3–53.3%) DEG were respectively up-regulated (FC ≥ 0.58) and downregulated (FC ≤ −0.58), in the 10 investigated MPM cell lines, by treatment.

**Figure 4:**
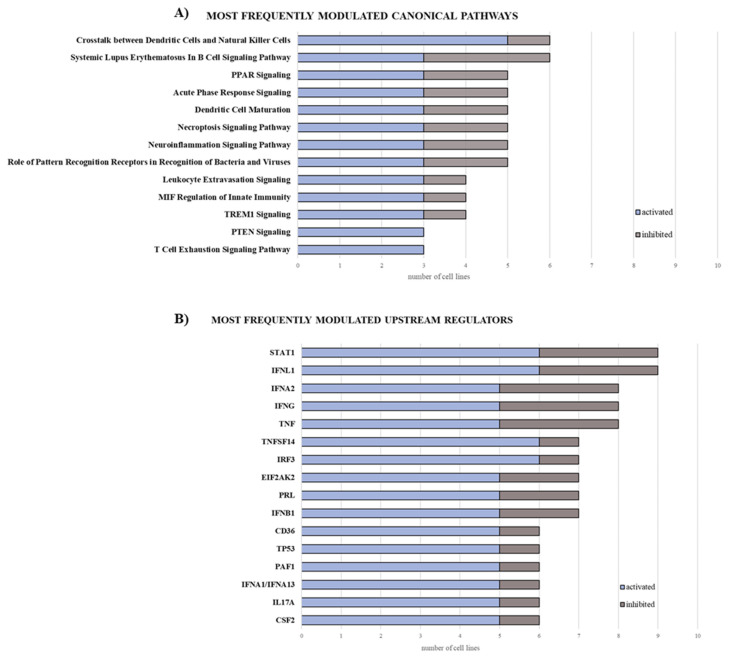
Bar graphs of the most frequently modulated canonical pathways and upstream regulators in MPM cell lines treated with guadecitabine. The gene expression profile of 10 MPM cell lines untreated or treated with guadecitabine was evaluated by NanoString nCounter profiler. Data analysis (log_2_ ratio of treated vs. untreated cell lines) was elaborated through IPA software, filtered by Z-score ≥ 2, and cumulated based on the frequency of modulation (activation and inhibition) for canonical pathways (**A**) and upstream regulators (**B**).

According to Ingenuity Pathway Analysis (IPA), 248 canonical pathways were modulated (Z-score ≥ 2 or Z-score ≤ −2) in treated compared to untreated cells, in at least one cell line (Table S3). Among the most frequently (in at least three cell lines) activated (Z-score ≥ 2) ones were those involved in the modulation of the immune response (e.g., crosstalk between dendritic cells and natural killer cells, dendritic cell maturation, acute phase response- and TREM1-signalling) with a frequency of activation ranging from 50% to 100% (Figure 4A; Table S3). In particular, the most frequently activated pathways, activation of the crosstalk between dendritic cells and natural killer (NK) cells signaling, include genes modulated by guadecitabine, such as CD209, CD28, CD40 and ligand, CD80, CD86, FAS and ligand, some classical HLA genes family members, IL-2/-4/-6, ICAM3, MICA, MICB, tumor necrosis factor (TNF), and toll-like receptor (TLR)-3/-4/-7/-9 among all (Table S3).

IPA upstream regulator analysis identified 660 upstream regulators responsible for changes in the expression profiles of at least one MPM cell line after guadecitabine treatment (Table S4). Among these, the most frequently (in at least three MPM cell lines) activated ones were mainly related to the pathways of interferon (IFN)-γ signaling (e.g., IFNL1, IRF1, IRF3, IFNA2, IFNB1, IFNA1/IFNA13) and TNF-α signaling (e.g., TNFSF14 and PRL), with a frequency of activation ranging from 62.5% to 85.71% (Figure 4B; Table S4).

Stemming from these results, we performed a subtype-specific investigation of the modulation of functional classes of immune-related genes, after guadecitabine treatment.

**Figure 5:**
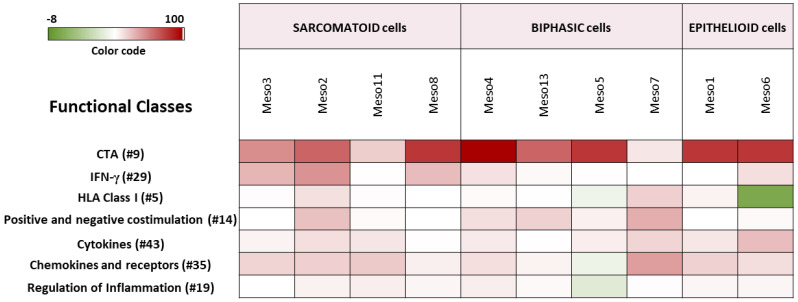
Modulation of specific functional classes of genes by guadecitabine treatment in MPM cell lines. A color scale was used to depict the predominant up- (red) or down- (green) regulation of genes belonging to the selected functional classes calculated as follow: (%up − %down) × (%up + %down)/100.

A strongly upregulated expression of multiple CTA genes was observed in all the three DHA-treated subtypes (Figure 5; Table S5). Comprehensively, a predominant upregulation of genes involved in several immune-related functional classes, such as IFN-γ and IFN- related genes, HLA class I, positive and negative co-stimulation, cytokines, chemokines and receptors and regulation of inflammation, was observed in the sarcomatoid MPM cell lines, compared to the lower effects observed in other investigated MPM subtypes (Figure 5; Table S5).

### 2.5. Validation of Gene Expression Profiling Changes by Guadecitabine

**Figure 6:**
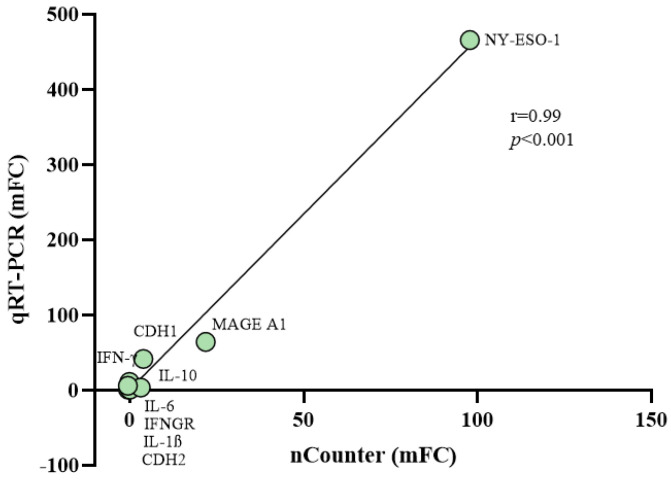
Correlation among changes in the expression values of selected genes after guadecitabine treatment from nCounter and quantitative real-time PCR analyses. Quantitative real-time PCR assays were performed in 10 untreated and guadecitabine-treated MPM cell lines to quantify the expression of 9 randomly selected genes. Mean values of FC (mFC) induced by guadecitabine in the constitutive levels of gene-specific mRNA expression were correlated to the mFC values obtained by the Nanostring analysis using Pearson’s correlation coefficient.

Quantitative real-time RT-PCR assays were performed to validate changes induced by guadecitabine in GEP of 10 MPM cell lines, by quantifying the expression of nine DEGs, randomly selected for their differential up- or down-regulation in all treated vs. untreated cell lines (Table S6). These included immunogenic CTA (i.e., NY-ESO-1 and MAGE-A1), immune-related genes (i.e., IFN-γ, IFNGR, interleukin (IL)-1β, IL-10, IL-6), and EMT markers (CDH1 and CDH2 cadherins). A significant correlation (r = 0.99; *p* < 0.001) was proved between changes (mean FC values) induced by guadecitabine in the constitutive levels of investigated genes expression, measured by quantitative real-time PCR or by nCounter multiplex gene expression analysis in the 10 MPM cell lines (Figure 6).

## 3. Discussion

Despite the accumulation of novel insights about mesothelioma biology and the considerable number of clinical investigations ongoing, MPM is still an aggressive cancer with a dismal prognosis and limited clinical benefit. ICI therapy represents an attractive strategy in the treatment of solid tumors; however, modest results have been obtained by single-agent or combined ICI-targeted immunotherapy, due to multiple mechanisms of resistance [18]. Since epigenetic modifications are known to contribute to the highly MPM immunosuppressive microenvironment [19], it is reasonable to explore the efficacy of epigenetic compounds in MPM, in the attempt to improve tumor immunogenicity, remodel tumor phenotype and provide a rationale for epigenetic-based strategies able to enhance the efficacy of immunotherapy. However, limited studies have been conducted to investigate the immunomodulatory activity of different classes of epigenetic drugs (i.e., HDACi or EZH2i) in MPM, compared to the well-characterized DHA; and even more limited are the studies that investigated the potential of combining DHA with these other epigenetic compounds in MPM [34,37,38]. This study aims to provide this missing information exploiting the availability of MPM cells of different histologic subtypes.

Along this line, the up-regulated expression of HLA class I antigens and of the co-stimulatory molecule, ICAM-1, induced by treatment with guadecitabine of MPM cells, alone or combined with VPA, SAHA, or EPZ-6438 to the DHA, identifies guadecitabine as the main driver of the immunomodulatory activity observed. This assertion is sustained by the increased levels of CTA expression observed in the three investigated MPM subtypes treated with guadecitabine alone or in combination. Accordingly, similar observations were recently reported using HDACi, at doses below optimal toxicity, that enhanced CTA expression in MPM epithelioid cells pre-treated with decitabine [34]. Therefore, among different available epigenetic drugs, guadecitabine represents a promising enhancer of immunogenicity of MPM cells and a potential inducer of increased immune cell recognition of tumor cells.

On this basis, we expanded the characterization of the immunomodulatory effect of guadecitabine on MPM cell lines through the nCounter methodology to comprehensively investigate changes in the expression of a wide number of genes related to tumor-, microenvironment- and immune response-signatures, after DHA treatment.

The demonstration that modulation of immune response was among the most frequently activated canonical pathway supports, in a broader view, the possibility of using guadecitabine to modulate MPM phenotype making it more immunogenic and possibly more responsive to immunotherapy. The activation of the crosstalk between dendritic cells and NK cells signaling was found to be the most frequently activated pathway in DHA-treated MPM cell lines. Previous data report how dendritic cells can trigger the NK-mediated innate immunity in HLA class I-negative cells, thus promoting NK cell killing and IFN-γ production in vivo [39]. From cytofluorimetric assays, we observed that the tested cell lines were positive for HLA class I antigens and guadecitabine up-regulated their surface expression in all investigated cell lines. In addition, the activation of the dendritic-NK cells crosstalk, induced by guadecitabine in the 50% of cell lines, was specifically observed in two biphasic and three sarcomatoid MPM cell lines (data not shown). Altogether these results support the hypothesis that guadecitabine could enhance both adaptive and non-adaptive immunity in the most aggressive MPM histological subtypes. Moreover, we found that the most frequently activated upstream regulator were mainly related to the IFN-γ signaling. This is of pivotal importance, given the involvement of IFN-γ in host–tumor interactions and in mechanisms of tumor resistance to therapeutic CTLA-4 blockade [40,41]. Moreover, guadecitabine exerted its strongest action in inducing IFN-related genes in the sarcomatoid phenotype, in which we observed, among others, a higher up-regulation of the ISG15 gene and its target proteins (e.g., MX1) required for NK cells proliferation, neutrophils chemotaxis, and IFN-γ-inducing cytokines production [42], compared to biphasic and epithelioid phenotypes (Table S5). These data confirm results about the epigenetic activation of the immune response through the IFN signaling, previously obtained in cell lines of different tumor histotypes [23,43], supporting the thesis that DHA could increase the immune response against MPM tumor cells, potentially also with sarcomatoid features. Our data also confirmed the immunomodulatory capabilities of guadecitabine, which induced a strong upregulation of CTA, especially CTAG1B (NY-ESO-1) and MAGE family members, in all the three histological subtypes of MPM, able to potentially boost both humoral and cell-mediated immune responses. Contrarily to the well-known activity of CTA, the role of TNF-dependent immune response in cancer progression is contradictory. Although some members of the superfamily can induce an immune response through the release of “danger signals”, other components seem to be pro-tumorigenic [44]. Based on our results, guadecitabine induced TNFRSF10A (in the three histotypes) and TNFRSF1B (in sarcomatoid and biphasic cell lines), sustaining cell death signals and apoptosis, but also CD70 (in seven mixed cell lines), which could potentially enhance the generation of cytolytic T cells and contribute to T cell activation. Moreover, a recent study reports the overexpression of the mesenchymal-associated TNFRSF1A to be strongly related to poor prognosis, and its knockdown to inhibit proliferation and migration of tumor cell lines in vitro [45]; also, it seems to induce the production of IL-17 by CD4+ T cells, recruiting myeloid cells and supporting tumor growth [46]. We registered the down-regulation of TNFRSF1A, especially in sarcomatoid and biphasic cell lines; this could be a mechanism, induced by guadecitabine, to impair tumor progression and to avoid the recruitment of immunosuppressive cells, that act as a barrier to cancer immunotherapy, also taking into consideration the down-regulation of the expression of IL17A-specific mRNA observed in the aforementioned phenotypes.

DHA were also demonstrated to sensitize tumors to ICI treatment through the up-regulated expression of immune checkpoint molecules [23,47]. In line with this, guadecitabine induced a strong up-regulation of CTLA-4 in sarcomatoid and biphasic cell lines, as well as of PD-L1 (CD274) in the biphasic phenotype, and of the co-stimulatory molecule CD40 in 50% of cell lines, making these molecules more targetable by immunotherapeutic strategies and supporting the use of epigenetic drugs in combination with immune checkpoint mAbs in the clinical setting (Table S5).

We reported the expression of the HLA-related molecule and migration inhibitory factor (MIF) ligand CD74, which represents an independent prognostic factor of survival for MPM patients, whatever the histological subtype, being, however, more expressed in the epithelial type; indeed, its low tumoral expression was associated with dismal prognosis [48]. Some studies also support the CD74 involvement in the prevention of the EMT process in other tumor histotypes [49,50,51]. CD74 was up-regulated by the treatment with guadecitabine only in cell lines of the sarcomatoid and biphasic phenotypes, encouraging the use of DHAs to revert mesenchymal features of MPM. Additionally, the induction of the expression of epithelial markers, such as E-cadherin, and the absent modulation of the mesenchymal markers, for example, N-cadherin and NCAM, observed after guadecitabine in sarcomatoid MPM cells, strengthens the interesting role of DNA hypomethylation in the modeling of the MPM aggressive phenotype, associated to the switch from epithelial markers to mesenchymal markers [9].

Although the number of investigated MPM cell lines was limited, the sensitivity to epigenetic drugs treatment, heterogeneously observed among MPM cell lines belonging to the three different subtypes, was more evident in the most aggressive sarcomatoid MPM subtype, supporting the need to develop personalized combination therapies to render these aggressive tumors more responsive to immunotherapy.

Comprehensively these studies laid the groundwork for planning further in vivo studies to demonstrate the potential of guadecitabine-based immunotherapeutic strategies for MPM treatment, considering also the influence of MPM subtypes in the epigenetic drugs’ sensitivity.

## 4. Materials and Methods

### 4.1. Cell Lines

Ten MPM cell lines were selected in order to represent the three main subtypes: the epithelioid Meso1 and Meso6, the biphasic Meso4, Meso5, Meso7 and Meso13, and the sarcomatoid Meso2, Meso3, Meso8 and Meso11. All cell lines were established by our group from pleural effusions of MPM patients deal with in the University Hospital of Siena, under approval by the Committee on Human Research. All cell lines were cultured in HAM’s F-12 medium (Biochrom, Berlin, Germany) supplemented with 10% heat-inactivated fetal bovine serum (Biochrom, Berlin, Germany), 2 mML-glutamine, and 100 µg/µL of penicillin/streptomycin (Biochrom, Berlin, Germany).

### 4.2. Monoclonal Antibodies and Epigenetic Drugs

PE mouse anti-human ICAM-1 clone 84H10 monoclonal antibody (mAb) was purchased from Beckman Coulter and alexafluor 488 mouse anti-human HLA-ABC (class I) clone W6/32 mAb was purchased from Biolegend. Guadecitabine was purchased from MedChemExpress LLC (Monmouth Junction, NJ, USA); HDAC inhibitors, VPA and SAHA, were purchased from Sigma Aldrich Corporation (St. Luis, MO, USA) and Cayman Chemical (Ann Arbor, MI, USA), respectively and the EZH2i EPZ-6438 was purchased from Selleck Chemicals (Houston, TX, USA).

### 4.3. In Vitro MPM Cells Treatment with Combined Epigenetic Drugs

MPM cell lines were seeded in T75 tissue culture flasks at a density of 1 × 10^6^/13 mL of complete medium (day 0) and treated with 1 µM guadecitabine [26] every 12 h for 2 days (day 1, day 2), or with 1mM VPA [34], 1.25 µM SAHA [34] or 1 µM EPZ-6438 [52] at day 3, and harvested at day 6. Combinatorial treatment of HDACi or EZH2i with guadecitabine maintained the same schedule of single treatments. Control cells were treated under similar experimental conditions in the absence of drugs. DNA hypomethylating activity of guadecitabine 1µM was confirmed by LINE-1 methylation assay performed on 10 MPM cells (Figure S1) as previously described [23].

### 4.4. Flow Cytometry Analysis

Cell surface expression of antigens on MPM cells was assessed by direct immunofluorescence staining followed by flow cytometry utilizing BD FACS Canto ™ (Beckman Coulter, Brea, CA, USA), according to the manufacturer’s instructions. Results were expressed as mean fluorescence intensity (MFI) of cell surface staining with respect to the unstained cells. The cell population was gated on FSC/SSC parameters and live cells were discriminated based on 7 AAD. Representative gating strategy was reported in Figure S2. Data were analyzed with the Kaluza^®^ Flow Analysis Software (Beckman Coulter, Brea, CA, USA). *p*-Value was calculated by paired Student *t*-test between values of MFI of surface molecules expressed on drugs-treated cells compared to untreated cells.

### 4.5. Quantitative Real-Time PCR Analysis

Total RNA was extracted by TRIzol reagent (Invitrogen, CA, USA), according to the manufacturer’s instruction and digested with RNAse-free DNAse (Roche Diagnostics GmbH, Mannheim, Germany). Synthesis of cDNA was performed on 2 µg of total RNA using M-MLV reverse transcriptase (Invitrogen, CA, USA) and random hexamer primers (Promega, Madison, WI, USA), according to the manufacturer’s instructions. Absolute quantifications were carried out using calibration curves, based on known scalar dose concentrations of recombinant plasmid DNA containing both the targets and the endogenous reference (i.e., β-actin) genes. For each experimental sample, the amount of target and of the endogenous reference was determined extrapolating values from the appropriate calibration curves (Figure S3). Then, the target amount is divided by the endogenous reference amount to obtain a normalized target value. QuantStudio™ 5 Real-Time PCR System (Applied Biosystems™, San Francisco, CA, USA) and its analyses software were used to conduct the quantitative real-time PCR analyses. The primers used for the quantitative real-time PCR analyses are listed in Table 1. Gene expression was considered positive if numbers of target gene/β-actin molecules were ≥10 × 10^−4^.

### 4.6. Nanostring Gene Expression Profiling

Total RNA (80ng) extracted from 10 MPM cell lines untreated or treated with guadecitabine was analyzed with the nCounter^®^ SPRINT Profiler (NanoString Technologies, Seattle, WA, USA). The PanCancer IO 360™ gene expression panel was used to evaluate simultaneously the number of 770 mRNA targets involved in the crucial interplay between the immune system, tumor and tumor microenvironment. Raw data were processed into a signature matrix using nSolver Analysis Software version 4.0 (NanoString Technologies Inc., Seattle, WA, USA). All Log2 ratios were generated by the nSolver^®^ Analysis Software.

### 4.7. Statistical Analysis

A paired Student *t*-test was used to calculate *p*-value for cytofluorimetric and molecular data and *p* < 0.05 was considered statistically significant. DEGs were selected if genes showed a Log2 ratio ≥ 0.58 or ≤−0.58 in guadecitabine-treated MPM lines vs. untreated ones and analyzed by IPA software to identify canonical pathways and upstream regulators modulated by treatment. In detail, modulation, activation, and inhibition scores of canonical pathways and upstream regulators were calculated counting the number of tumor cell lines for which a specific pathway was modulated (Z-score ≥ 2 or Z-score ≤ −2), activated (Z-score ≥ 2), or inhibited (Z-score ≤ −2), by guadecitabine compared to baseline. % of activation or inhibition was calculated as the ratio between the activation and inhibition frequency, respectively, and the modulation score.

The modulation of genes belonging to a specific functional class was calculated as follow:

[(%up − %down) × (%up + %down)/100]. Correlation between mFC in the expression of selected genes, measured by nCounter assay and quantitative real-time PCR analyses, was evaluated by Pearson’s correlation coefficient (r).

Statistical analyses were carried out by GraphPad Prism 7.05 (GraphPad Software Inc., San Diego, CA, USA).

## Figures and Tables

**Table 1:** Primer sequences sets for quantitative real-time PCR analysis.

	Forward Sequence	Reverse Sequence
NY-ESO-1	5′-TGCTTGAGTTCTACCTCGCCA-3′	5′-TATGTTGCCGGACACAGTGAA-3′
MAGE-A1	5′-GCCAAGCACCTCTTGTATCCTG-3′	5′-GGAGCAGAAAACCAACCAAATC-3′
MAGE-A3	5′-TGTCGTCGGAAATTGGCAGTAT-3′	5′-CAAAGACCAGCTGCAAGGAACT-3′
CDH1	5′-AGAGACTGGGTTATTCCTCC-3′	5′-GGATTTGATCTGAACCAGGT-3′
CDH2	5′-CCTTTCAAACACAGCCACGG-3′	5′-TGTTTGGGTCGGTCTGGATG-3′
IL-1β	5′-ACTTGTTCTTTGAAGCTGATGGC-3′	5′-CTGTAGTGGTGGTCGGAGATTC-3′
IFN-γ	5′-CAGGTCATTCAGATGTAGCGGAT-3′	5′-ATGTCTTCCTTGATGGTCTCCAC-3′
IFNGR	5′-CATCACGTCATACCAGCCATTT-3′	5′-ATGTCTTCCTTGATGGTCTCCAC-3′
IL-6	5′-AACCTGAACCTTCCAAAGATGG-3′	5′-TCTGGCTTGTTCCTCACTACT-3′
IL-10	5′-CGGCGCTGTCATCGATTT-3′	5′-TTAAAGGCATTCTTCACCTGCTC-3′

## Data Availability

The data that support the findings of this study are available from the corresponding author upon reasonable request.

## Supplementary Materials

The following are available online at https://www.mdpi.com/article/10.3390/epigenomes5040027/s1, Figure S1: Methylation status of LINE-1 in 10 MPM cell lines treated with guadecitabine; Figure S2: Flow cytometry analysis of MPM cell lines untreated or treated with epigenetic drugs; Figure S3: Real-time RT-PCR amplification settings; Table S1: Flow cytometry analysis of MPM cell lines treated with epigenetic drugs; Table S2: Quantitative RT-PCR analysis of MPM cell lines treated with epigenetic drugs; Table S3: Canonical pathways modulated in MPM cell lines by guadecitabine treatment; Table S4: Upstream regulators modulated in MPM cell lines by guadecitabine treatment; Table S5: Gene specific expression nCounter data in MPM cell lines treated with guadecitabine vs untreated ones; Table S6: Real-Time PCR and nCounter expression values of selected genes in MPM cell lines treated with guadecitabine vs untreated ones.
