# Supplementary material for: Epigenetic Immune Remodeling of Mesothelioma Cells: A New Strategy to Improve the Efficacy of Immunotherapy
Source: Epigenomes. 2021 Dec 14;5(4):27. doi: 10.3390/epigenomes5040027 (PMC8715476; doi:10.3390/epigenomes5040027)
Supplement: Supplementary file 1 [file epigenomes-05-00027-s001.zip › Figure S1.pdf]

Supplemental Figure S1. Methylation status of LINE-1 in 10 MPM cell lines treated with guadecitabine

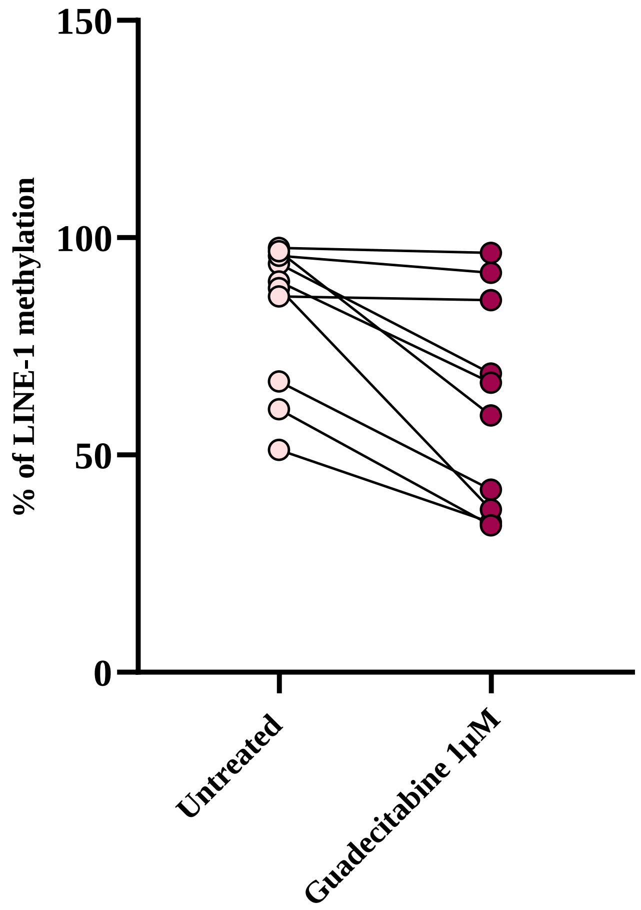

Genomic DNA was extracted from 10 MPM cell lines treated with 1 µM of guadecitabine. Real-time qMSP analyses of LINE-1 were performed on bisulfite modified genomic DNA using methylated- or unmethylated-specific primer pairs.

Data are reported as % of LINE-1 methylation in guadecitabine treated vs untreated cells.
