# Supplementary material for: Epigenetic Immune Remodeling of Mesothelioma Cells: A New Strategy to Improve the Efficacy of Immunotherapy
Source: Epigenomes. 2021 Dec 14;5(4):27. doi: 10.3390/epigenomes5040027 (PMC8715476; doi:10.3390/epigenomes5040027)

Supplemental Figure S2. Flow cytometry analysis of MPM cell lines untreated or treated with epigenetic drugs

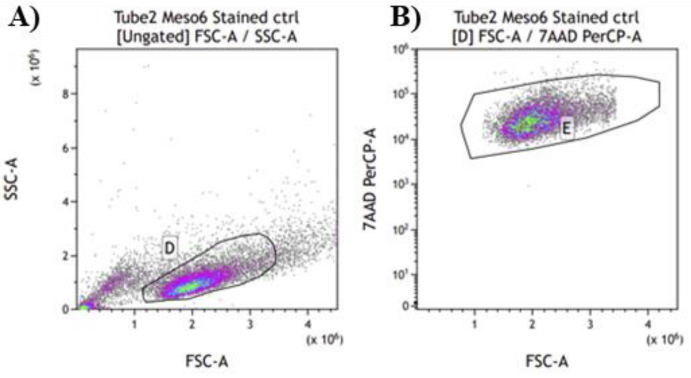

Overview of a representative gating strategy, in flow cytometry, delineated by gating of total cell population via on FSC/SSC (**A**). Based on live cells gating discriminate on 7AAD (**B**), ICAM-1 and HLA class I positivity was determined.

Flow cytometry analysis of changes induced by different epigenetic drugs in HLA class I and ICAM-1 molecules expression. MPM cell lines untreated or treated with guadecitabine, VPA, SAHA, EPZ-6438, or guadecitabine-based combinations were incubated with (**C**) anti-human HLA class I or (**D**) anti-human ICAM-1 mAbs and studied by flow cytometry. Histogram profile of each investigated antigen, from one representative experiment, is shown for all investigated MPM cell lines untreated or treated with different epigenetic drugs alone or in combinations.

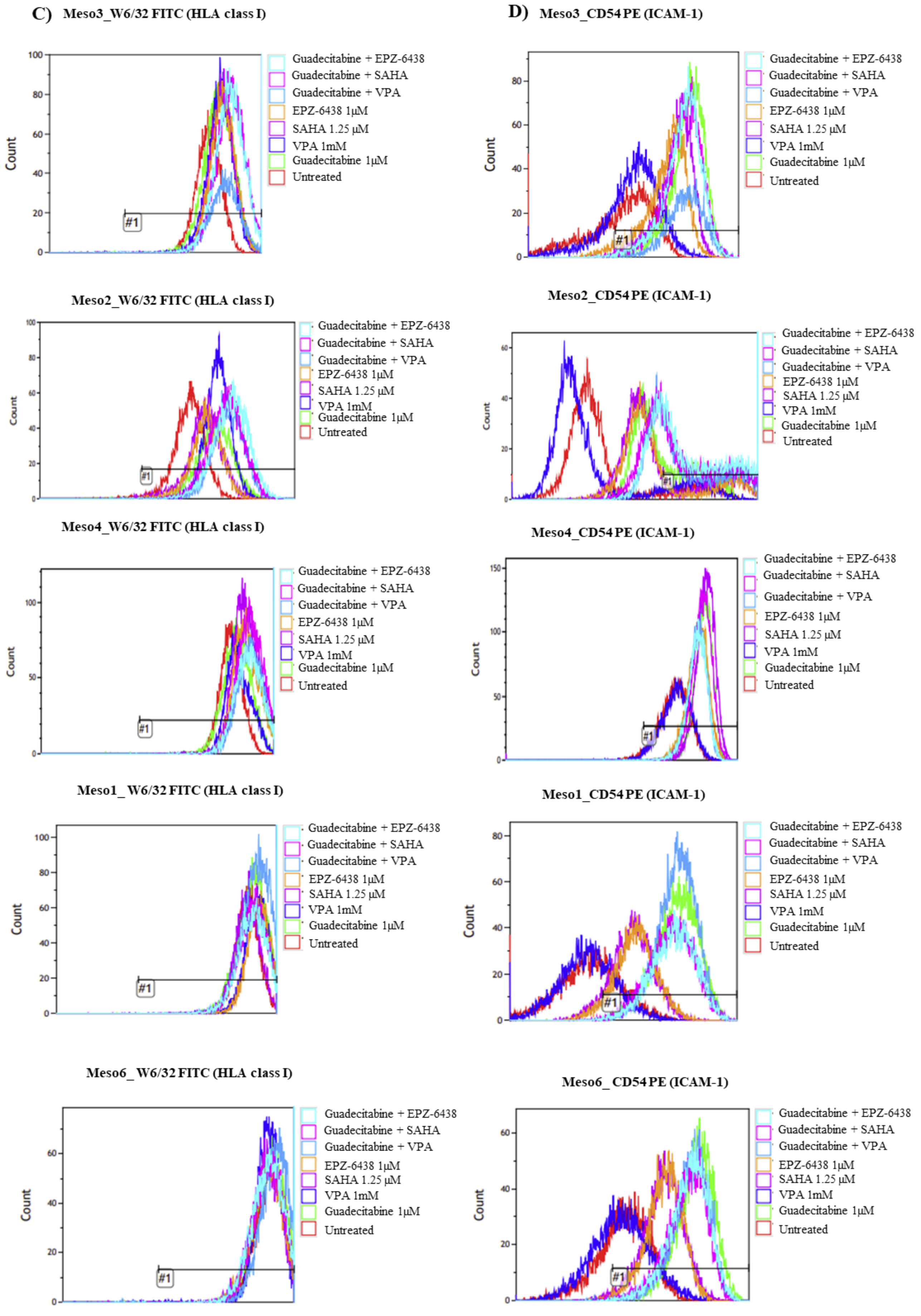

Supplement: Supplementary file 1 [file epigenomes-05-00027-s001.zip › Figure S2.pdf]
