# Supplementary material for: Epigenetic Immune Remodeling of Mesothelioma Cells: A New Strategy to Improve the Efficacy of Immunotherapy
Source: Epigenomes. 2021 Dec 14;5(4):27. doi: 10.3390/epigenomes5040027 (PMC8715476; doi:10.3390/epigenomes5040027)

Supplemental Figure S3

Real-time RT-PCR amplification settings:

a) Amplification plots of the calibration curve for endogenous reference and targets;

b) Standard curves generated from known quantities of the target genes.

a) β-actin b)

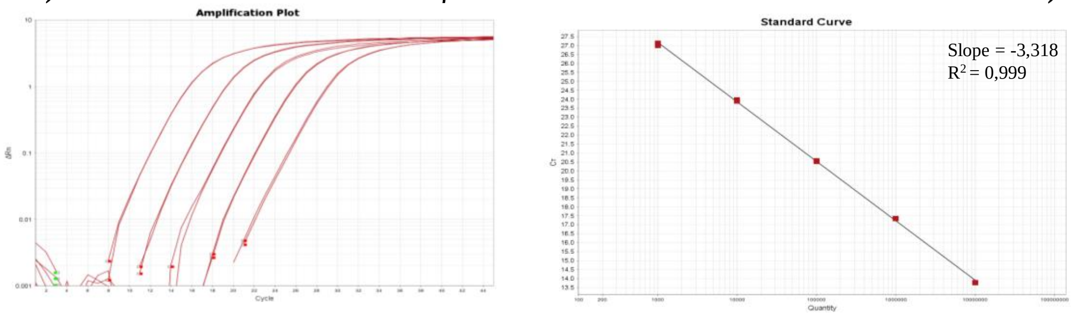

a) MAGE-A1 b)

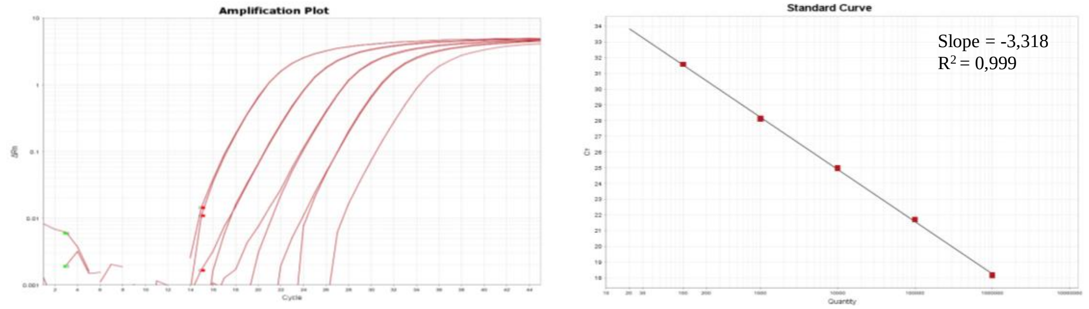

MAGE-A3

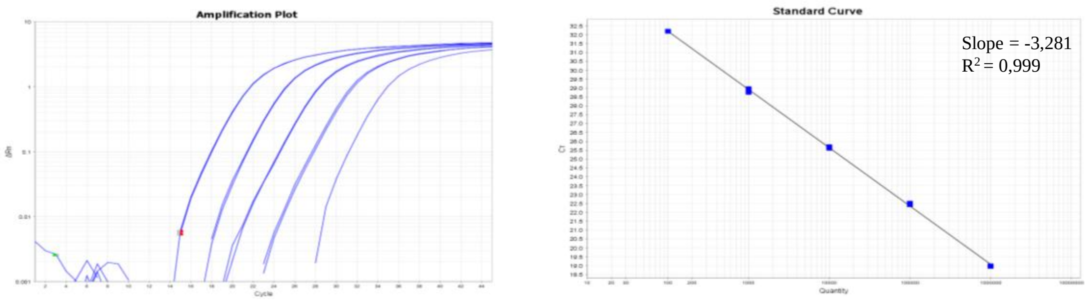

CDH1

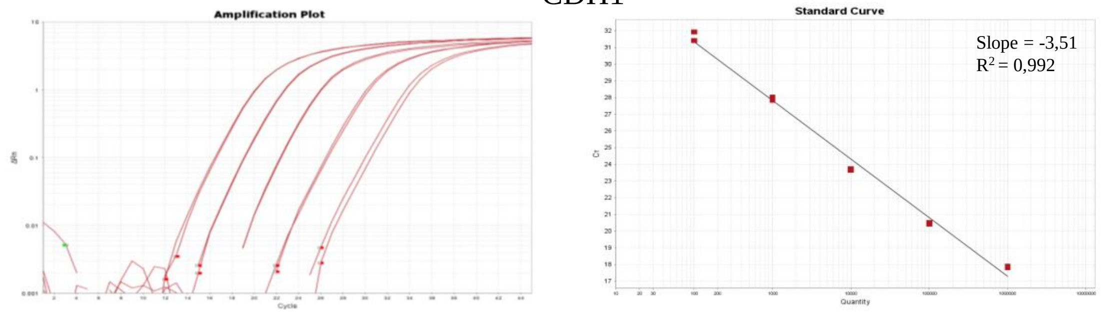

NY-ESO-1

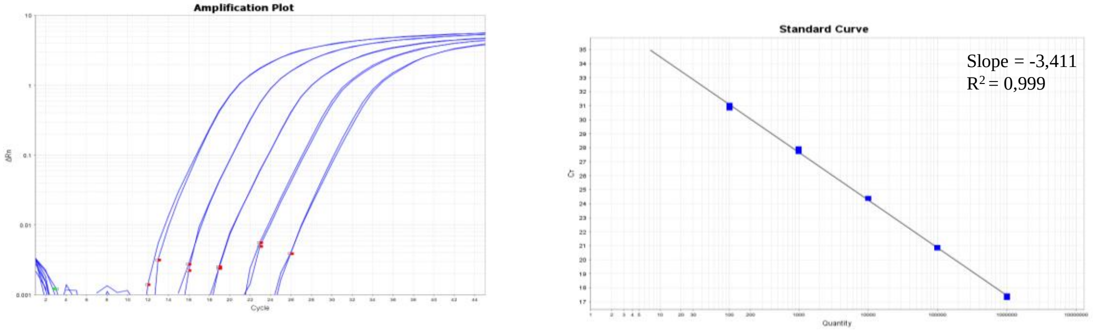

CDH2

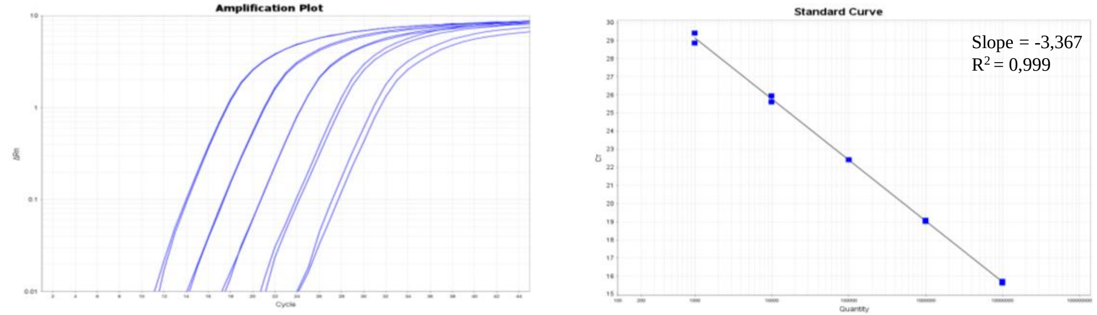

Supplement: Supplementary file 1 [file epigenomes-05-00027-s001.zip › Figure S3.pdf]
