# Supplementary material for: Epigenetic Immune Remodeling of Mesothelioma Cells: A New Strategy to Improve the Efficacy of Immunotherapy
Source: Epigenomes. 2021 Dec 14;5(4):27. doi: 10.3390/epigenomes5040027 (PMC8715476; doi:10.3390/epigenomes5040027)
Supplement: Supplementary file 1 [file epigenomes-05-00027-s001.zip › Table S1.pdf]

Supplemental Table S1. Flow cytometry analysis of MPM cell lines treated with epigenetic drugs

HLA class I MFI	Untreated	Guadecitabine 1µM	VPA 1mM	SAHA 1.25 µM	EPZ-6438 1µM	Guadecitabine + VPA	Guadecitabine+ SAHA	Guadecitabine + EPZ-6438
Meso3 (sarcomatoid)								
Mean ±SD	190.29 (±6.10) [a]	226.54 (±3.12)	171.79 (±13.58)	186.68 (±1.69)	207.14 (±3.18)	190.87 (±5.71)	225.89 (±3.37)	199.59 (±13.97)
Paired Student t Test		***0.018***	***0.089***	***0.315***	***0.039***	***0.478***	***0.059***	***0.172***
Meso2 (sarcomatoid)								
Mean ±SD	35.20 (±0.57)	55.95 (±3.61)	55.90 (±1.84)	39.50 (±0.57)	39.60 (±0.00)	44.45 (±0.49)	54.85 (±1.48)	62.85 (±0.78)
Paired Student t Test		***0.045***	***0.026***	***0.059***	***0.029***	***0.026***	***0.011***	***0.011***
Meso4 (biphasic)								
Mean ±SD	193.75 (±2.19)	228.82 (±8.93)	171.73 (±8.03)	231.08 (±12.76)	198.25 (±21.99)	193.70 (±16.97)	210.55 (±11.81)	197.25 (±6.86)
Paired Student t Test		***0.043***	***0.059***	***0.117***	***0.401***	***0.498***	***0.122***	***0.241***
Meso1 (epithelioid)								
Mean ±SD	420.85 (±21.28)	569.15 (±6.58)	470.62 (±78.46)	508.29 (±6.35)	463.54 (±11.52)	560.99 (±15.96)	543.43 (±25.21)	541.37 (±17.21)
Paired Student t Test		***0.042***	***0.217***	***0.070***	***0.158***	***0.059***	***0.083***	***0.071***
Meso6 (epithelioid)								
Mean ±SD	308.56 (±310.76)	550.62 (±162.33)	297.86 (±258.49)	253.47 (±198.00)	192.78 (±140.50)	376.14 (±15.77)	330.06 (±96.14)	327.25 (±4.71)
Paired Student t Test		***0.130***	***0.410***	***0.308***	***0.256***	***0.400***	***0.455***	***0.473***

ICAM-1 MFI	Untreated	Guadecitabine 1µM	VPA 1mM	SAHA 1.25 µM	EPZ-6438 1µM	Guadecitabine + VPA	Guadecitabine+ SAHA	Guadecitabine + EPZ-6438
Meso3 (sarcomatoid)								
Mean ±SD	104.78 (±5.02) [a]	309.80 (±3.22)	152.15 (±13.58)	133.56 (±3.78)	148.82 (±1.28)	460.44 (±12.73)	376.10 (±6.12)	316.74 (±1.20)
Paired Student t Test		***0.002***	***0.040***	***0.068***	***0.019***	***0.005***	***0.009***	***0.004***
Meso2 (sarcomatoid)								
Mean ±SD	17.15 (±5.58)	45.58 (±26.86)	26.75 (±0.59)	34.61 (±4.89)	30.05 (±1.88)	46.90 (±20.96)	48.25 (±12.09)	59.55 (±6.85)
Paired Student t Test		***0.155***	***0.112***	***0.009***	***0.064***	***0.112***	***0.047***	***0.065***
Meso4 (biphasic)								
Mean ±SD	1078.85 (±21.94)	1243.76 (±12.12)	1096.50 (±136.99)	1613.50 (±103.33)	1217.60 (±0.01)	998.17 (±43.35)	1335.19 (±62.83)	1091.58 (±128.15)
Paired Student t Test		***0.013***	***0.432***	***0.034***	***0.035***	***0.059***	***0.073***	***0.447***
Meso1 (epithelioid)								
Mean ±SD	43.50 (±5.39)	343.93 (±26.84)	39.69 (±2.19)	47.80 (±8.54)	47.05 (±2.71)	396.76 (±1.02)	316.76 (±96.11)	375.16 (±14.99)
Paired Student t Test		***0.016***	***0.171***	***0.369***	***0.157***	***0.004***	***0.073***	***0.007***
Meso6 (epithelioid)								
Mean ±SD	110.36 (±30.45)	460.38 (±34.70)	105.62 (±13.37)	106.74 (±9.50)	88.39 (±7.13)	361.18 (±5.38)	298.82 (±51.96)	318.02 (±17.76)
Paired Student t Test		***0.003***	***0.452***	***0.460***	***0.280***	***0.032***	***0.026***	***0.052***

ICAM-1 % of positive cells	Untreated	Guadecitabine 1µM	VPA 1mM	SAHA 1.25 µM	EPZ-6438 1µM	Guadecitabine + VPA	Guadecitabine+ SAHA	Guadecitabine + EPZ-6438
Meso3 (sarcomatoid)								

Mean ±SD	83.65 (±20.06) [b]	96.20 (±4.78)	82.83 (±24.03)	90.19 (±10.06)	90.63 (±8.97)	98.55 (±1.82)	97.80 (±3.21)	97.77 (±3.18)
Paired Student t Test		***0.145***	***0.378***	***0.187***	***0.195***	***0.146***	***0.142***	***0.142***
Meso2 (sarcomatoid)								
Mean ±SD	16.46 (±3.22)	36.77 (±8.20)	13.62 (±6.93)	19.86 (±9.62)	17.37 (±4.63)	34.24 (±11.97)	36.61 (±8.03)	41.76 (±5.97)
Paired Student t Test		***0.013***	***0.187***	***0.244***	***0.228***	***0.042***	***0.011***	***0.003***
Meso4 (biphasic)								
Mean ±SD	98.81 (±2.07)	98.90 (±1.81)	95.13 (±8.34)	92.85 (±12.38)	95.14 (±8.42)	96.50 (±6.07)	96.05 (±6.84)	96.25 (±6.41)
Paired Student t Test		***0.292***	***0.209***	***0.211***	***0.211***	***0.211***	***0.211***	***0.208***
Meso1 (epithelioid)								
Mean ±SD	93.20 (±6.95)	99.20 (±0.53)	92.73 (±5.35)	92.47 (±4.74)	93.03 (±8.11)	99.87 (±0.06)	99.50 (±0.35)	99.43 (±0.46)
Paired Student t Test		***0.124***	***0.333***	***0.367***	***0.435***	***0.118***	***0.120***	***0.119***
Meso6 (epithelioid)								
Mean ±SD	98.33 (±2.12)	99.54 (±0.45)	98.36 (±2.23)	97.41 (±2.93)	98.27 (±0.97)	99.67 (±0.11)	99.57 (±0.15)	99.48 (±0.39)
Paired Student t Test		***0.168***	***0.371***	***0.094***	***0.471***	***0.189***	***0.195***	***0.185***

[a] values are reported as mean values of MFI

[b] values are reported as % of positive cells
