# Supplementary material for: Epigenetic Immune Remodeling of Mesothelioma Cells: A New Strategy to Improve the Efficacy of Immunotherapy
Source: Epigenomes. 2021 Dec 14;5(4):27. doi: 10.3390/epigenomes5040027 (PMC8715476; doi:10.3390/epigenomes5040027)
Supplement: Supplementary file 1 [file epigenomes-05-00027-s001.zip › Table S2.pdf]

Supplemental Table S2. Quantitative RT-PCR analysis of MPM cell lines treated with epigenetic drugs

NY-ESO-1	Untreated	Guadecitabine 1µM	VPA 1mM	SAHA 1.25 µM	EPZ-6438 1µM	Guadecitabine + VPA	Guadecitabine+ SAHA	Guadecitabine + EPZ-6438
Meso3 (sarcomatoid)								
Mean ± SD	8.76E-06 (±3.47E-06) [a]	2.18E-02 (±9.18E-03)	1.76E-05 (±5.70E-06)	1.15E-05 (±3.60E-06)	7.40E-06 (±3.32E-06)	3.44E-02 (±1.25E-02)	2.36E-02 (±5.48E-03)	2.31E-02 (±3.00E-03)
Paired Student t Test		***0.027***	***0.013***	***0.063***	***0.112***	***0.021***	***0.009***	***0.003***
Meso2 (sarcomatoid)								
Mean ± SD	1.57E-05 (±1.42E-05)	3.00E-03 (±2.57E-04)	8.30E-06 (±4.04E-06)	2.44E-05 (±3.16E-05)	5.66E-06 (±7.21E-07)	5.42E-03 (±1.95E-03)	2.88E-03 (±3.43E-04)	3.61E-03 (±1.10E-04)
Paired Student t Test		***0.001***	***0.170***	***0.379***	***0.169***	***0.020***	***0.002***	***0.000***
Meso4 (biphasic)								
Mean ± SD	6.56E-05 (±2.89E-05)	3.66E-02 (±2.10E-03)	8.40E-05 (±2.27E-05)	1.09E-04 (±4.24E-05)	1.05E-04 (±2.47E-05)	5.66E-02 (±1.44E-02)	2.90E-02 (±4.79E-03)	3.34E-02 (±1.32E-02)
Paired Student t Test		***0.001***	***0.230***	***0.189***	***0.133***	***0.011***	***0.005***	***0.024***
Meso1 (epithelioid)								
Mean ± SD	9.44E-06 (±3.33E-06)	1.02E-02 (±3.57E-03)	1.20E-05 (±2.46E-06)	1.04E-05 (±4.93E-06)	1.06E-05 (±6.07E-06)	2.58E-02 (±8.94E-03)	1.16E-02 (±3.34E-03)	1.08E-02 (±1.22E-03)
Paired Student t Test		***0.019***	***0.024***	***0.304***	***0.274***	***0.019***	***0.013***	***0.002***
Meso6 (epithelioid)								
Mean ± SD	9.32E-06 (±7.73E-07)	6.59E-03 (±3.11E-03)	1.15E-05 (±4.41E-06)	8.18E-06 (±1.10E-06)	7.53E-06 (±3.16E-06)	1.64E-02 (±9.57E-03)	6.03E-03 (±1.99E-03)	6.87E-03 (±2.90E-03)
Paired Student t Test		***0.034***	***0.221***	***0.028***	***0.222***	***0.049***	***0.017***	***0.027***

MAGE-A1	Untreated	Guadecitabine 1µM	VPA 1mM	SAHA 1.25 µM	EPZ-6438 1µM	Guadecitabine + VPA	Guadecitabine+ SAHA	Guadecitabine + EPZ-6438
Meso3 (sarcomatoid)								
Mean ± SD	2.01E-02 (±4.90E-03)	4.41E-02 (±1.80E-02)	4.10E-02 (±1.22E-02)	2.33E-02 (±8.97E-03)	1.97E-02 (±1.28E-02)	7.61E-02 (±3.68E-02)	4.86E-02 (±1.41E-02)	4.59E-02 (±9.03E-03)
Paired Student t Test		***0.043***	***0.019***	***0.153***	***0.466***	***0.047***	***0.017***	***0.005***
Meso2 (sarcomatoid)								
Mean ± SD	1.59E-02 (±7.66E-03)	2.98E-02 (±1.28E-02)	2.11E-02 (±1.16E-02)	1.80E-02 (±6.10E-03)	1.37E-02 (±5.77E-03)	4.00E-02 (±1.93E-02)	2.58E-02 (±1.24E-02)	2.80E-02 (±1.18E-02)
Paired Student t Test		***0.022***	***0.144***	***0.223***	***0.105***	***0.035***	***0.034***	***0.020***
Meso4 (biphasic)								
Mean ± SD	6.82E-05 (±1.41E-05)	5.93E-03 (±1.83E-03)	1.65E-04 (±1.18E-04)	6.82E-05 (±4.34E-05)	5.77E-05 (±2.20E-05)	1.75E-02 (±5.15E-03)	5.59E-03 (±1.63E-03)	6.18E-03 (±2.10E-03)
Paired Student t Test		***0.016***	***0.144***	***0.500***	***0.262***	***0.014***	***0.014***	***0.019***
Meso1 (epithelioid)								
Mean ± SD	7.81E-06 (±1.72E-06)	2.12E-03 (±9.13E-04)	1.49E-05 (±1.48E-05)	1.11E-05 (±2.49E-06)	8.05E-06 (±2.52E-06)	8.14E-03 (±3.26E-03)	2.17E-03 (±7.13E-04)	2.24E-03 (±4.43E-04)
Paired Student t Test		***0.028***	***0.265***	***0.087***	***0.439***	***0.025***	***0.017***	***0.006***
Meso6 (epithelioid)								
Mean ± SD	3.11E-04 (±1.16E-04)	8.28E-04 (±2.54E-04)	1.86E-04 (±1.21E-04)	1.86E-04 (±1.29E-04)	1.51E-04 (±1.19E-04)	3.15E-03 (±2.18E-03)	6.51E-04 (±2.92E-04)	8.69E-04 (±2.97E-04)
Paired Student t Test		***0.068***	***0.228***	***0.233***	***0.178***	***0.083***	***0.142***	***0.071***

MAGE-A3	Untreated	Guadecitabine 1µM	VPA 1mM	SAHA 1.25 µM	EPZ-6438 1µM	Guadecitabine + VPA	Guadecitabine+ SAHA	Guadecitabine + EPZ-6438
Meso3 (sarcomatoid)								
Mean ± SD	8.29E-02 (±1.91E-02)	1.73E-01 (±7.00E-02)	1.29E-01 (±5.39E-02)	9.74E-02 (±4.34E-02)	8.43E-02 (±4.47E-02)	2.25E-01 (±1.12E-01)	1.62E-01 (±4.93E-02)	1.71E-01 (±4.96E-02)
Paired Student t Test		***0.046***	***0.074***	***0.215***	***0.362***	***0.059***	***0.023***	***0.019***
Meso2 (sarcomatoid)								

Mean ± SD	1.51E-02 (±6.42E-03)	3.37E-02 (±1.56E-02)	1.75E-02 (±7.53E-03)	1.93E-02 (±2.54E-03)	1.36E-02 (±4.76E-03)	4.41E-02 (±2.34E-02)	2.81E-02 (±1.30E-02)	3.33E-02 (±1.44E-02)
Paired Student t Test		***0.038***	***0.172***	***0.184***	***0.131***	***0.049***	***0.041***	***0.031***
Meso4 (biphasic)								
Mean ± SD	4.86E-04 (±4.03E-04)	2.65E-02 (±1.31E-02)	6.28E-04 (±2.88E-04)	4.24E-04 (±2.93E-04)	5.33E-04 (±3.83E-04)	4.89E-02 (±1.53E-02)	2.48E-02 (±1.07E-02)	2.67E-02 (±1.08E-02)
Paired Student t Test		***0.029***	***0.182***	***0.163***	***0.332***	***0.003***	***0.033***	***0.019***
Meso1 (epithelioid)								
Mean ± SD	6.78E-02 (±2.85E-02)	7.24E-02 (±2.61E-02)	6.16E-02 (±2.02E-02)	6.33E-02 (±2.42E-02)	6.23E-02 (±2.44E-02)	1.04E-01 (±5.14E-02)	6.99E-02 (±2.80E-02)	7.35E-02 (±2.71E-02)
Paired Student t Test		***0.266***	***0.184***	***0.117***	***0.090***	***0.057***	***0.313***	***0.026***
Meso6 (epithelioid)								
Mean ± SD	3.55E-02 (±6.66E-04)	4.35E-02 (±9.37E-03)	2.41E-02 (±2.73E-03)	3.03E-02 (±6.34E-03)	2.96E-02 (±3.40E-03)	5.31E-02 (±2.43E-02)	3.43E-02 (±1.13E-02)	4.06E-02 (±8.06E-03)
Paired Student t Test		***0.132***	***0.011***	***0.137***	***0.052***	***0.165***	***0.437***	***0.188***

**CDH1**	**Untreated**	**Guadecitabine 1µM**	**VPA 1mM**	**SAHA 1.25 µM**	**EPZ-6438 1µM**	**Guadecitabine + VPA**	**Guadecitabine+ SAHA**	**Guadecitabine + EPZ-6438**
Meso3 (sarcomatoid)								
Mean ± SD	5.47E-05 (±4.64E-06)	6.84E-04 (±4.22E-04)	8.77E-05 (±1.91E-05)	5.94E-05 (±5.83E-06)	5.35E-05 (±3.07E-06)	2.35E-03 (±1.42E-03)	7.10E-04 (±4.75E-04)	7.26E-04 (±3.73E-04)
Paired Student t Test		***0.062***	***0.048***	***0.134***	***0.411***	***0.054***	***0.071***	***0.045***
Meso2 (sarcomatoid)								
Mean ± SD	1.10E-05 (±8.59E-06)	6.20E-04 (±5.19E-05)	2.53E-05 (±3.21E-05)	1.82E-05 (±2.31E-05)	1.16E-05 (±1.00E-05)	1.75E-03 (±8.69E-04)	6.24E-04 (±1.43E-04)	8.55E-04 (±1.70E-04)
Paired Student t Test		***0.001***	***0.203***	***0.243***	***0.274***	***0.037***	***0.008***	***0.006***
Meso4 (biphasic)								
Mean ± SD	9.68E-02 (±2.67E-02)	1.14E-01 (±3.10E-02)	4.72E-02 (±3.33E-02)	1.02E-01 (±4.77E-03)	9.23E-02 (±2.01E-02)	6.90E-02 (±3.03E-02)	1.13E-01 (±3.46E-02)	1.07E-01 (±2.91E-02)
Paired Student t Test		***0.138***	***0.010***	***0.375***	***0.251***	***0.040***	***0.097***	***0.271***
Meso1 (epithelioid)								
Mean ± SD	7.81E-02 (±8.47E-03)	7.25E-02 (±7.33E-03)	6.77E-02 (±1.37E-02)	7.14E-02 (±2.55E-03)	7.69E-02 (±3.24E-03)	7.44E-02 (±5.06E-03)	6.60E-02 (±1.03E-02)	8.12E-02 (±4.19E-03)
Paired Student t Test		***0.298***	***0.250***	***0.154***	***0.438***	***0.185***	***0.188***	***0.171***
Meso6 (epithelioid)								
Mean ± SD	5.89E-02 (±6.01E-03)	8.18E-02 (±8.05E-03)	6.31E-02 (±1.92E-02)	6.02E-02 (±7.96E-03)	6.36E-02 (±2.02E-03)	1.10E-01 (±3.35E-02)	8.38E-02 (±8.85E-03)	8.68E-02 (±1.21E-03)
Paired Student t Test		***0.004***	***0.320***	***0.188***	***0.179***	***0.042***	***0.003***	***0.006***

**CDH2**	**Untreated**	**Guadecitabine 1µM**	**VPA 1mM**	**SAHA 1.25 µM**	**EPZ-6438 1µM**	**Guadecitabine + VPA**	**Guadecitabine+ SAHA**	**Guadecitabine + EPZ-6438**
Meso3 (sarcomatoid)								
Mean ± SD	2.47E-02 (±1.45E-02)	2.87E-02 (±1.77E-02)	4.14E-02 (±2.48E-02)	2.76E-02 (±1.44E-02)	2.69E-02 (±1.83E-02)	5.90E-02 (±3.85E-02)	3.48E-02 (±2.10E-02)	2.39E-02 (±1.25E-02)
Paired Student t Test		***0.098***	***0.055***	***0.046***	***0.300***	***0.066***	***0.060***	***0.281***
Meso2 (sarcomatoid)								
Mean ± SD	4.94E-02 (±3.90E-02)	4.39E-02 (±3.29E-02)	5.38E-02 (±4.52E-02)	5.20E-02 (±3.84E-02)	3.57E-02 (±2.67E-02)	5.08E-02 (±2.84E-02)	4.58E-02 (±3.31E-02)	3.61E-02 (±2.61E-02)
Paired Student t Test		***0.131***	***0.340***	***0.396***	***0.121***	***0.439***	***0.224***	***0.114***
Meso4 (biphasic)								
Mean ± SD	2.47E-02 (±1.79E-02)	1.65E-02 (±1.19E-02)	2.60E-02 (±1.83E-02)	2.33E-02 (±1.63E-02)	2.35E-02 (±1.64E-02)	2.73E-02 (±1.75E-02)	1.49E-02 (±1.04E-02)	1.61E-02 (±1.09E-02)
Paired Student t Test		***0.142***	***0.430***	***0.294***	***0.205***	***0.114***	***0.077***	***0.120***
Meso1 (epithelioid)								
Mean ± SD	2.04E-06 (±7.14E-07)	1.66E-05 (±1.23E-05)	2.98E-06 (±1.16E-06)	1.66E-06 (±8.34E-07)	2.40E-06 (±1.48E-06)	1.07E-04 (±7.38E-05)	2.31E-05 (±1.96E-05)	4.50E-05 (±2.73E-05)
Paired Student t Test		***0.081***	***0.090***	***0.031***	***0.293***	***0.065***	***0.097***	***0.054***

Meso6 (epithelioid)								
Mean ± SD	1.77E-06 (±2.90E-07)	6.05E-05 (±4.46E-05)	3.36E-06 (±6.52E-07)	1.91E-06 (±5.90E-07)	2.72E-06 (±1.36E-06)	1.59E-04 (±1.20E-04)	4.54E-05 (±2.94E-05)	9.51E-05 (±6.26E-05)
Paired Student t Test		***0.075***	***0.018***	***0.390***	***0.194***	***0.075***	***0.062***	***0.061***

[a] Data are reported as the number of immune molecules-specific mRNA normalized to the number of β-actin molecules, obtained by qRT-PCR analysis
