# Supplementary material for: Epigenetic Immune Remodeling of Mesothelioma Cells: A New Strategy to Improve the Efficacy of Immunotherapy
Source: Epigenomes. 2021 Dec 14;5(4):27. doi: 10.3390/epigenomes5040027 (PMC8715476; doi:10.3390/epigenomes5040027)
Supplement: Supplementary file 1 [file epigenomes-05-00027-s001.zip › Table S4.pdf]

Supplemental Table S4. Upstream regulators modulated in MPM cell lines by guadecitabine treatment

Upstream regulators	Modulation score[a]	Activation score[b] (%)[c]	Inhibition score[d] (%)[e]
IFNL1	9	6(67.0)	3(33.0)
STAT1	9	6(67.0)	3(33.0)
IRF3	7	6(86.0)	1(14.0)
TNFSF14	7	6(86.0)	1(14.0)
TNF	8	5(62.5)	3(37.5)
IFNG	8	5(62.5)	3(37.5)
IFNA2	8	5(62.5)	3(37.5)
IFNB1	7	5(71.0)	3(37.5)
PRL	7	5(71.0)	2(29.0)
EIF2AK2	7	5(71.0)	2(29.0)
CSF2	6	5(83.0)	1(29.0)
IL17A	6	5(83.0)	1(17.0)
IFNA1/IFNA13	6	5(83.0)	1(17.0)
PAF1	6	5(83.0)	1(17.0)
TP53	6	5(83.0)	1(17.0)
CD36	6	5(83.0)	1(17.0)
IRF1	7	4(57.0)	3(17.0)
P38 MAPK	6	4(67.0)	2(17.0)
IRF7	6	4(67.0)	2(17.0)
RAF1	6	4(67.0)	2(43.0)
IL1B	6	4(67.0)	2(43.0)
RNY3	6	4(67.0)	2(43.0)
FOXO1	5	4(80.0)	1(33.0)
TLR3	5	4(80.0)	1(33.0)
FOXO3	5	4(80.0)	1(33.0)
TLR4	5	4(80.0)	1(33.0)
FOXL2	5	4(80.0)	1(33.0)
RELA	5	4(80.0)	1(33.0)
ERK1/2	5	4(80.0)	1(33.0)
CXCL12	5	4(80.0)	1(33.0)
IL27	5	4(80.0)	1(33.0)
CD40LG	5	4(80.0)	1(20.0)
IL1A	5	4(80.0)	1(20.0)
TLR7	5	4(80.0)	1(20.0)
TLR9	5	4(80.0)	1(20.0)
IL18	5	4(80.0)	1(20.0)
TLR2	5	4(80.0)	1(20.0)
IKBKB	5	4(80.0)	1(20.0)
OSM	5	4(80.0)	1(20.0)
CHD1	5	4(80.0)	1(20.0)
CAMP	5	4(80.0)	1(20.0)
IL17F	5	4(80.0)	1(20.0)
APP	5	4(80.0)	1(20.0)
SMPD1	5	4(80.0)	1(20.0)
ELF4	4	4(100.0)	0(0.0)
BMP2	4	4(100.0)	0(0.0)
RC3H1	7	3(43.0)	4(57.0)
SMARCA4	6	3(50.0)	3(50.0)
E2F1	6	3(50.0)	3(50.0)
NKX2-3	5	3(60.0)	2(40.0)
TRADD	5	3(60.0)	2(40.0)
IFNL4	5	3(60.0)	2(40.0)
FOXM1	4	3(75.0)	1(25.0)
IL2	4	3(75.0)	1(25.0)
TGM2	4	3(75.0)	1(25.0)
EP300	4	3(75.0)	1(25.0)
PI3K (complex)	4	3(75.0)	1(25.0)
F2	4	3(75.0)	1(25.0)
IL15	4	3(75.0)	1(25.0)
IL1	4	3(75.0)	1(25.0)
TNFSF11	4	3(75.0)	1(25.0)
IFNA21	4	3(75.0)	1(25.0)
IFNA5	4	3(75.0)	1(25.0)
IFNA10	4	3(75.0)	1(25.0)
IFNA7	4	3(75.0)	1(25.0)
IFNA14	4	3(75.0)	1(25.0)
IFNA6	4	3(75.0)	1(25.0)
IFNA8	4	3(75.0)	1(25.0)
IFNA16	4	3(75.0)	1(25.0)
CDKN2A	4	3(75.0)	1(25.0)
miR-34a-5p (and other miRNAs w/seed GGCAGUG)	4	3(75.0)	1(25.0)
IFI16	4	3(75.0)	1(25.0)
MIF	4	3(75.0)	1(25.0)
SPI1	4	3(75.0)	1(25.0)
PTGS2	4	3(75.0)	1(25.0)
PML	4	3(75.0)	1(25.0)
ORMDL3	4	3(75.0)	1(25.0)
CREBBP	3	3(100.0)	0(0.0)
RNASE1	3	3(100.0)	0(0.0)
miR-16-5p (and other miRNAs w/seed AGCAGCA)	3	3(100.0)	0(0.0)

TIFA	3	3(100.0)	0(0.0)
IL1RAP	3	3(100.0)	0(0.0)
S100A7	3	3(100.0)	0(0.0)
LGALS8	3	3(100.0)	0(0.0)
IL36A	3	3(100.0)	0(0.0)
PRKD1	3	3(100.0)	0(0.0)
mir-15	3	3(100.0)	0(0.0)
RNF31	3	3(100.0)	0(0.0)
IL17R	3	3(100.0)	0(0.0)
miR-199a-5p (and other miRNAs w/seed CCAGUGU)	7	2(29.0)	5(71.0)
TGFB1	5	2(40.0)	3(60.0)
CCN1	5	2(40.0)	3(60.0)
MAPK1	5	2(40.0)	3(60.0)
PDGF BB	4	2(50.0)	2(50.0)
CD40	4	2(50.0)	2(50.0)
HGF	4	2(50.0)	2(50.0)
EGFR	4	2(50.0)	2(50.0)
EBI3	4	2(50.0)	2(50.0)
IFNE	4	2(50.0)	2(50.0)
TP63	4	2(50.0)	2(50.0)
STAT3	4	2(50.0)	2(50.0)
HIF1A	4	2(50.0)	2(50.0)
FOS	4	2(50.0)	2(50.0)
AGT	4	2(50.0)	2(50.0)
TBK1	4	2(50.0)	2(50.0)
GPER1	4	2(50.0)	2(50.0)
COL18A1	4	2(50.0)	2(50.0)
miR-155-5p (miRNAs w/seed UAAUGCU)	4	2(50.0)	2(50.0)
miR-21-5p (and other miRNAs w/seed AGCUUAU)	4	2(50.0)	2(50.0)
TNFSF10	4	2(50.0)	2(50.0)
CEBPB	3	2(67.0)	1(33.0)
VEGFA	3	2(67.0)	1(33.0)
AR	3	2(67.0)	1(33.0)
CD3	3	2(67.0)	1(33.0)
JUN	3	2(67.0)	1(33.0)
CHUK	3	2(67.0)	1(33.0)
EGF	3	2(67.0)	1(33.0)
SP1	3	2(67.0)	1(33.0)
NFKB1	3	2(67.0)	1(33.0)
PRKCD	3	2(67.0)	1(33.0)
MAP3K14	3	2(67.0)	1(33.0)
MAPK14	3	2(67.0)	1(33.0)
EPAS1	3	2(67.0)	1(33.0)
IFNA4	3	2(67.0)	1(33.0)
HMGB1	3	2(67.0)	1(33.0)
IRF9	3	2(67.0)	1(33.0)
mir-17	3	2(67.0)	1(33.0)
IL32	3	2(67.0)	1(33.0)
PF4	3	2(67.0)	1(33.0)
CGAS	3	2(67.0)	1(33.0)
TNFSF12	3	2(67.0)	1(33.0)
STING1	3	2(67.0)	1(33.0)
PLG	3	2(67.0)	1(33.0)
TLR8	3	2(67.0)	1(33.0)
VCAN	3	2(67.0)	1(33.0)
DDX58	3	2(67.0)	1(33.0)
IL36B	3	2(67.0)	1(33.0)
FOXO4	2	2(100.0)	0(0.0)
CXCL8	2	2(100.0)	0(0.0)
ITGAL	2	2(100.0)	0(0.0)
MAP2K1	2	2(100.0)	0(0.0)
POU2F2	2	2(100.0)	0(0.0)
RNASE2	2	2(100.0)	0(0.0)
IL23	2	2(100.0)	0(0.0)
PARP9	2	2(100.0)	0(0.0)
CIC	2	2(100.0)	0(0.0)
SP110	2	2(100.0)	0(0.0)
mir-155	2	2(100.0)	0(0.0)
mir-10	2	2(100.0)	0(0.0)
miR-17-5p (and other miRNAs w/seed AAAGUGC)	2	2(100.0)	0(0.0)
miR-199a-3p (and other miRNAs w/seed CAGUAGU)	2	2(100.0)	0(0.0)
miR-29b-3p (and other miRNAs w/seed AGCACCA)	2	2(100.0)	0(0.0)
miR-203a-3p (and other miRNAs w/seed UGAAAUG)	2	2(100.0)	0(0.0)
mir-145	2	2(100.0)	0(0.0)
miR-125b-5p (and other miRNAs w/seed CCCUGAG)	2	2(100.0)	0(0.0)
miR-141-3p (and other miRNAs w/seed AACACUG)	2	2(100.0)	0(0.0)
mir-34	2	2(100.0)	0(0.0)
SAHM1	2	2(100.0)	0(0.0)
TLR5	2	2(100.0)	0(0.0)
SCAVENGER receptor CLASS A	2	2(100.0)	0(0.0)
mir-30 (includes others)	2	2(100.0)	0(0.0)
mir-1	2	2(100.0)	0(0.0)
RUNX3	2	2(100.0)	0(0.0)

NUP98-DDX10	2	2(100.0)	0(0.0)
IL17C	2	2(100.0)	0(0.0)
TREM1	2	2(100.0)	0(0.0)
SPDEF	2	2(100.0)	0(0.0)
B4GALT6	2	2(100.0)	0(0.0)
NUPR1	2	2(100.0)	0(0.0)
TNFRSF1A	2	2(100.0)	0(0.0)
RAD21	2	2(100.0)	0(0.0)
ETS2	2	2(100.0)	0(0.0)
WWC1	2	2(100.0)	0(0.0)
PPRC1	2	2(100.0)	0(0.0)
EZH2	2	2(100.0)	0(0.0)
STAT6	2	2(100.0)	0(0.0)
IL24	2	2(100.0)	0(0.0)
ZBTB7A	2	2(100.0)	0(0.0)
PRDM1	2	2(100.0)	0(0.0)
SAFB2	2	2(100.0)	0(0.0)
CALCA	2	2(100.0)	0(0.0)
FAS	2	2(100.0)	0(0.0)
IL1RN	7	1(14.0)	6(86.0)
PKM	5	1(20.0)	4(80.0)
CTNNB1	4	1(25.0)	3(75.0)
NLRC5	4	1(25.0)	3(75.0)
JAK	4	1(25.0)	3(75.0)
SYVN1	4	1(25.0)	3(75.0)
KDM3A	4	1(25.0)	3(75.0)
NPC2	4	1(25.0)	3(75.0)
CBX5	4	1(25.0)	3(75.0)
ETV6-RUNX1	4	1(25.0)	3(75.0)
miR-146a-5p (and other miRNAs w/seed GAGAACU)	4	1(25.0)	3(75.0)
BTK	4	1(25.0)	3(75.0)
USP18	4	1(25.0)	3(75.0)
MEOX2	4	1(25.0)	3(75.0)
SAFB	4	1(25.0)	3(75.0)
SOCS3	4	1(25.0)	3(75.0)
RAC1	3	1(33.0)	2(67.0)
HRAS	3	1(33.0)	2(67.0)
ERBB2	3	1(33.0)	2(67.0)
SPP1	3	1(33.0)	2(67.0)
JAK1	3	1(33.0)	2(67.0)
IFNK	3	1(33.0)	2(67.0)
TGFB3	3	1(33.0)	2(67.0)
SRC	3	1(33.0)	2(67.0)
ECSIT	3	1(33.0)	2(67.0)
JAG1	3	1(33.0)	2(67.0)
PGR	3	1(33.0)	2(67.0)
NPC1	3	1(33.0)	2(67.0)
IL37	3	1(33.0)	2(67.0)
HNF1A-AS1	3	1(33.0)	2(67.0)
JAG2	3	1(33.0)	2(67.0)
CNOT7	3	1(33.0)	2(67.0)
TNFRSF18	3	1(33.0)	2(67.0)
S100A6	3	1(33.0)	2(67.0)
PPP2R5C	3	1(33.0)	2(67.0)
IKZF1	3	1(33.0)	2(67.0)
SOCS1	3	1(33.0)	2(67.0)
IRF4	3	1(33.0)	2(67.0)
mir-146	3	1(33.0)	2(67.0)
SMC3	2	1(50.0)	1(50.0)
SRC (family)	2	1(50.0)	1(50.0)
SMAD4	2	1(50.0)	1(50.0)
ATM	2	1(50.0)	1(50.0)
MITF	2	1(50.0)	1(50.0)
FLT1	2	1(50.0)	1(50.0)
PTP4A1	2	1(50.0)	1(50.0)
IKBKE	2	1(50.0)	1(50.0)
C5	2	1(50.0)	1(50.0)
CCL5	2	1(50.0)	1(50.0)
IL4	2	1(50.0)	1(50.0)
LDL	2	1(50.0)	1(50.0)
ETS1	2	1(50.0)	1(50.0)
FGF2	2	1(50.0)	1(50.0)
RAS	2	1(50.0)	1(50.0)
EDN1	2	1(50.0)	1(50.0)
IL5	2	1(50.0)	1(50.0)
NRG1	2	1(50.0)	1(50.0)
F3	2	1(50.0)	1(50.0)
ICAM1	2	1(50.0)	1(50.0)
EGR1	2	1(50.0)	1(50.0)
CCND1	2	1(50.0)	1(50.0)
ELK1	2	1(50.0)	1(50.0)
PRKCA	2	1(50.0)	1(50.0)
IGF1R	2	1(50.0)	1(50.0)

PFKFB3	2	1(50.0)	1(50.0)
PPARD	2	1(50.0)	1(50.0)
EWSR1	2	1(50.0)	1(50.0)
IL6	2	1(50.0)	1(50.0)
IL12 (complex)	2	1(50.0)	1(50.0)
IL7	2	1(50.0)	1(50.0)
MYD88	2	1(50.0)	1(50.0)
IL12 (family)	2	1(50.0)	1(50.0)
BRD4	2	1(50.0)	1(50.0)
SAA	2	1(50.0)	1(50.0)
TICAM1	2	1(50.0)	1(50.0)
RETN	2	1(50.0)	1(50.0)
H2AB3 (includes others)	2	1(50.0)	1(50.0)
mir-8	2	1(50.0)	1(50.0)
AURKB	2	1(50.0)	1(50.0)
miR-450a-5p (and other miRNAs w/seed UUUGCGA)	2	1(50.0)	1(50.0)
LATS1	2	1(50.0)	1(50.0)
PKNOX2	2	1(50.0)	1(50.0)
PLA2G2A	2	1(50.0)	1(50.0)
mir-181	2	1(50.0)	1(50.0)
S100A8	2	1(50.0)	1(50.0)
PGF	2	1(50.0)	1(50.0)
PLCE1	2	1(50.0)	1(50.0)
C5AR1	2	1(50.0)	1(50.0)
SREBF1	2	1(50.0)	1(50.0)
F2R	2	1(50.0)	1(50.0)
SYK	2	1(50.0)	1(50.0)
ALB	2	1(50.0)	1(50.0)
ABL1	2	1(50.0)	1(50.0)
MAP2K4	2	1(50.0)	1(50.0)
MET	2	1(50.0)	1(50.0)
MAML1	2	1(50.0)	1(50.0)
LEP	2	1(50.0)	1(50.0)
FN1	2	1(50.0)	1(50.0)
MAVS	2	1(50.0)	1(50.0)
TCIM	2	1(50.0)	1(50.0)
TF	2	1(50.0)	1(50.0)
IL22	2	1(50.0)	1(50.0)
OSCAR	2	1(50.0)	1(50.0)
TNFSF9	2	1(50.0)	1(50.0)
FBXO42	2	1(50.0)	1(50.0)
IFIH1	2	1(50.0)	1(50.0)
NAMPT	2	1(50.0)	1(50.0)
S100A9	2	1(50.0)	1(50.0)
TLR7/8	2	1(50.0)	1(50.0)
BAPTA-AM	2	1(50.0)	1(50.0)
SAMHD1	2	1(50.0)	1(50.0)
DACH1	2	1(50.0)	1(50.0)
GPS2	2	1(50.0)	1(50.0)
IL7R	2	1(50.0)	1(50.0)
IL13	1	1(100.0)	0(0.0)
FOXC2	1	1(100.0)	0(0.0)
TIMP1	1	1(100.0)	0(0.0)
TCF3-PBX1	1	1(100.0)	0(0.0)
IGF1	1	1(100.0)	0(0.0)
GATA2	1	1(100.0)	0(0.0)
STAT	1	1(100.0)	0(0.0)
GLI1	1	1(100.0)	0(0.0)
WWTR1	1	1(100.0)	0(0.0)
CD44	1	1(100.0)	0(0.0)
TCR	1	1(100.0)	0(0.0)
KITLG	1	1(100.0)	0(0.0)
TNFSF13B	1	1(100.0)	0(0.0)
CD86	1	1(100.0)	0(0.0)
SOX7	1	1(100.0)	0(0.0)
GAST	1	1(100.0)	0(0.0)
EGLN	1	1(100.0)	0(0.0)
miR-873-5p (and other miRNAs w/seed CAGGAAC)	1	1(100.0)	0(0.0)
mir-199	1	1(100.0)	0(0.0)
miR-18a-5p (and other miRNAs w/seed AAGGUGC)	1	1(100.0)	0(0.0)
miR-1-3p (and other miRNAs w/seed GGAAUGU)	1	1(100.0)	0(0.0)
mir-148	1	1(100.0)	0(0.0)
ID4	1	1(100.0)	0(0.0)
mir-214	1	1(100.0)	0(0.0)
miR-26a-5p (and other miRNAs w/seed UCAAGUA)	1	1(100.0)	0(0.0)
miR-24-3p (and other miRNAs w/seed GGCUCAG)	1	1(100.0)	0(0.0)
ZGPAT	1	1(100.0)	0(0.0)
RNF216	1	1(100.0)	0(0.0)
miR-221-3p (and other miRNAs w/seed GCUACAU)	1	1(100.0)	0(0.0)
miR-218-5p (and other miRNAs w/seed UGUGCUU)	1	1(100.0)	0(0.0)
miR-291a-3p (and other miRNAs w/seed AAGUGCU)	1	1(100.0)	0(0.0)
ZFP36	1	1(100.0)	0(0.0)
RBL2	1	1(100.0)	0(0.0)

miR-451a (and other miRNAs w/seed AACCGUU)	1	1(100.0)	0(0.0)
APC	1	1(100.0)	0(0.0)
CTLA4	1	1(100.0)	0(0.0)
CCN2	1	1(100.0)	0(0.0)
KL	1	1(100.0)	0(0.0)
NCOA2	1	1(100.0)	0(0.0)
IL1RL2	1	1(100.0)	0(0.0)
PNN	1	1(100.0)	0(0.0)
USP7	1	1(100.0)	0(0.0)
IL36G	1	1(100.0)	0(0.0)
LTB4R	1	1(100.0)	0(0.0)
PTEN	1	1(100.0)	0(0.0)
GJA1	1	1(100.0)	0(0.0)
SPHK1	1	1(100.0)	0(0.0)
PRKN	1	1(100.0)	0(0.0)
UBE2I	1	1(100.0)	0(0.0)
CDKN2B-AS1	1	1(100.0)	0(0.0)
TAL1	1	1(100.0)	0(0.0)
ADORA3	1	1(100.0)	0(0.0)
MGAT3	1	1(100.0)	0(0.0)
XIAP	1	1(100.0)	0(0.0)
KLF4	1	1(100.0)	0(0.0)
FCER1G	1	1(100.0)	0(0.0)
TNFRSF8	1	1(100.0)	0(0.0)
MAP3K11	1	1(100.0)	0(0.0)
BIRC2	1	1(100.0)	0(0.0)
TPR	1	1(100.0)	0(0.0)
FCER2	1	1(100.0)	0(0.0)
ACTN4	1	1(100.0)	0(0.0)
TRPM2	1	1(100.0)	0(0.0)
mir-218	1	1(100.0)	0(0.0)
RFXAP	1	1(100.0)	0(0.0)
KAT5	1	1(100.0)	0(0.0)
SATB2	1	1(100.0)	0(0.0)
CST5	1	1(100.0)	0(0.0)
PLAU	1	1(100.0)	0(0.0)
MAP3K1	1	1(100.0)	0(0.0)
SMAD1	1	1(100.0)	0(0.0)
BSG	1	1(100.0)	0(0.0)
MAP2K7	1	1(100.0)	0(0.0)
E2f	1	1(100.0)	0(0.0)
THPO	1	1(100.0)	0(0.0)
LMNA	1	1(100.0)	0(0.0)
TBX5	1	1(100.0)	0(0.0)
RXRA	1	1(100.0)	0(0.0)
ZEB1	1	1(100.0)	0(0.0)
LUCAT1	1	1(100.0)	0(0.0)
TOPBP1	1	1(100.0)	0(0.0)
LIN9	1	1(100.0)	0(0.0)
mir-25	1	1(100.0)	0(0.0)
mir-135	1	1(100.0)	0(0.0)
HAVCR1	1	1(100.0)	0(0.0)
ANLN	1	1(100.0)	0(0.0)
HMOX1	1	1(100.0)	0(0.0)
BCL6	1	1(100.0)	0(0.0)
PP1	1	1(100.0)	0(0.0)
mir-26	1	1(100.0)	0(0.0)
MACROH2A1	1	1(100.0)	0(0.0)
LILRB4	1	1(100.0)	0(0.0)
ATG7	1	1(100.0)	0(0.0)
ZBED2	1	1(100.0)	0(0.0)
RCAN1	1	1(100.0)	0(0.0)
CITED2	1	1(100.0)	0(0.0)
NGLY1	1	1(100.0)	0(0.0)
PDCD4	1	1(100.0)	0(0.0)
miR-296-5p (miRNAs w/seed GGGCCCC)	1	1(100.0)	0(0.0)
SMAD7	1	1(100.0)	0(0.0)
NEUROG1	1	1(100.0)	0(0.0)
mir-373	1	1(100.0)	0(0.0)
HLX	1	1(100.0)	0(0.0)
mir-515	1	1(100.0)	0(0.0)
NPM1	1	1(100.0)	0(0.0)
FBXW7	1	1(100.0)	0(0.0)
SFTPA1	1	1(100.0)	0(0.0)
NF2	1	1(100.0)	0(0.0)
mir-133	1	1(100.0)	0(0.0)
FOXP3	1	1(100.0)	0(0.0)
TLR1	1	1(100.0)	0(0.0)
BTRC	1	1(100.0)	0(0.0)
mir-204	1	1(100.0)	0(0.0)
COL1A1	1	1(100.0)	0(0.0)
POU2AF1	1	1(100.0)	0(0.0)
IKK (complex)	1	1(100.0)	0(0.0)

ELANE	1	1(100.0)	0(0.0)
IL31	1	1(100.0)	0(0.0)
AREG	1	1(100.0)	0(0.0)
MAPKAPK2	1	1(100.0)	0(0.0)
TNFRSF14	1	1(100.0)	0(0.0)
AHR	1	1(100.0)	0(0.0)
IL19	1	1(100.0)	0(0.0)
NFAT5	1	1(100.0)	0(0.0)
BCL11B	1	1(100.0)	0(0.0)
HNF1B	1	1(100.0)	0(0.0)
MYC	4	0(0.0)	4(100.0)
IKZF3	4	0(0.0)	4(100.0)
SP3	3	0(0.0)	3(100.0)
GNA12	3	0(0.0)	3(100.0)
YBX1	3	0(0.0)	3(100.0)
NCOA3	3	0(0.0)	3(100.0)
EIF4G1	3	0(0.0)	3(100.0)
TEAD4	3	0(0.0)	3(100.0)
TEAD1	3	0(0.0)	3(100.0)
E2F6	3	0(0.0)	3(100.0)
OGA	3	0(0.0)	3(100.0)
HDAC2	3	0(0.0)	3(100.0)
NR3C1	3	0(0.0)	3(100.0)
NLRP12	3	0(0.0)	3(100.0)
PAEP	3	0(0.0)	3(100.0)
ITGA6	2	0(0.0)	2(100.0)
PLD1	2	0(0.0)	2(100.0)
OIP5-AS1	2	0(0.0)	2(100.0)
CD28	2	0(0.0)	2(100.0)
MYB	2	0(0.0)	2(100.0)
IFNAR2	2	0(0.0)	2(100.0)
CD2	2	0(0.0)	2(100.0)
KDM8	2	0(0.0)	2(100.0)
PCGEM1	2	0(0.0)	2(100.0)
NFE2L2	2	0(0.0)	2(100.0)
SP4	2	0(0.0)	2(100.0)
FGF7	2	0(0.0)	2(100.0)
IRF5	2	0(0.0)	2(100.0)
STAT5a/b	2	0(0.0)	2(100.0)
ATF4	2	0(0.0)	2(100.0)
KAT2B	2	0(0.0)	2(100.0)
USF2	2	0(0.0)	2(100.0)
FGFR1	2	0(0.0)	2(100.0)
TEAD2	2	0(0.0)	2(100.0)
CCAR2	2	0(0.0)	2(100.0)
MRTFB	2	0(0.0)	2(100.0)
RBL1	2	0(0.0)	2(100.0)
mir-29	2	0(0.0)	2(100.0)
IL6R	2	0(0.0)	2(100.0)
SNHG20	2	0(0.0)	2(100.0)
CCAT2	2	0(0.0)	2(100.0)
TAB1	2	0(0.0)	2(100.0)
CLDN7	2	0(0.0)	2(100.0)
NFKBIA	2	0(0.0)	2(100.0)
MAP3K7	2	0(0.0)	2(100.0)
DCLK1	2	0(0.0)	2(100.0)
CHRNA3	2	0(0.0)	2(100.0)
GRP	2	0(0.0)	2(100.0)
TAP1	2	0(0.0)	2(100.0)
LY6E	2	0(0.0)	2(100.0)
MERTK	2	0(0.0)	2(100.0)
DEPTOR	2	0(0.0)	2(100.0)
IL4I1	2	0(0.0)	2(100.0)
PDCD1	2	0(0.0)	2(100.0)
TEAD3	2	0(0.0)	2(100.0)
LIPG	2	0(0.0)	2(100.0)
CRNDE	2	0(0.0)	2(100.0)
FLI1	2	0(0.0)	2(100.0)
LATS2	2	0(0.0)	2(100.0)
PIM2	1	0(0.0)	1(100.0)
STAT2	1	0(0.0)	1(100.0)
Z-LLL-CHO	1	0(0.0)	1(100.0)
CREB1	1	0(0.0)	1(100.0)
IGFBP2	1	0(0.0)	1(100.0)
EIF4E	1	0(0.0)	1(100.0)
STAT4	1	0(0.0)	1(100.0)
BMP7	1	0(0.0)	1(100.0)
TP73	1	0(0.0)	1(100.0)
TGFA	1	0(0.0)	1(100.0)
WT1	1	0(0.0)	1(100.0)
MYCN	1	0(0.0)	1(100.0)
PCDH11Y	1	0(0.0)	1(100.0)
MED15	1	0(0.0)	1(100.0)

LINC01139	1	0(0.0)	1(100.0)
HNF4A	1	0(0.0)	1(100.0)
ERK	1	0(0.0)	1(100.0)
PI3K (family)	1	0(0.0)	1(100.0)
IL3	1	0(0.0)	1(100.0)
AKT1	1	0(0.0)	1(100.0)
REL	1	0(0.0)	1(100.0)
MMP1	1	0(0.0)	1(100.0)
TRAF2	1	0(0.0)	1(100.0)
PRKCE	1	0(0.0)	1(100.0)
IL21	1	0(0.0)	1(100.0)
CCL11	1	0(0.0)	1(100.0)
TAC1	1	0(0.0)	1(100.0)
POU5F1	1	0(0.0)	1(100.0)
EPO	1	0(0.0)	1(100.0)
MMP2	1	0(0.0)	1(100.0)
NFATC1	1	0(0.0)	1(100.0)
ATF2	1	0(0.0)	1(100.0)
PTAFR	1	0(0.0)	1(100.0)
TP53COR1	1	0(0.0)	1(100.0)
MAP2K1/2	1	0(0.0)	1(100.0)
PPP1R1B	1	0(0.0)	1(100.0)
ELAVL1	1	0(0.0)	1(100.0)
ANGPT2	1	0(0.0)	1(100.0)
CD5	1	0(0.0)	1(100.0)
TNFSF15	1	0(0.0)	1(100.0)
PRKCB	1	0(0.0)	1(100.0)
CYTOR	1	0(0.0)	1(100.0)
IFNW1	1	0(0.0)	1(100.0)
RUNX2	1	0(0.0)	1(100.0)
ILK	1	0(0.0)	1(100.0)
TYROBP	1	0(0.0)	1(100.0)
TSLP	1	0(0.0)	1(100.0)
ACKR3	1	0(0.0)	1(100.0)
M344	1	0(0.0)	1(100.0)
BMP4	1	0(0.0)	1(100.0)
SMAD2	1	0(0.0)	1(100.0)
ITGB3	1	0(0.0)	1(100.0)
MUC4	1	0(0.0)	1(100.0)
TCF7L2	1	0(0.0)	1(100.0)
A2M	1	0(0.0)	1(100.0)
ITGAV	1	0(0.0)	1(100.0)
E2F2	1	0(0.0)	1(100.0)
MRTFA	1	0(0.0)	1(100.0)
SOX2	1	0(0.0)	1(100.0)
NEDD9	1	0(0.0)	1(100.0)
NORAD	1	0(0.0)	1(100.0)
NSUN6	1	0(0.0)	1(100.0)
RABL6	1	0(0.0)	1(100.0)
CRP	1	0(0.0)	1(100.0)
KDM5B	1	0(0.0)	1(100.0)
miR-23a-3p (and other miRNAs w/seed UCACAUU)	1	0(0.0)	1(100.0)
CREM	1	0(0.0)	1(100.0)
KLF2	1	0(0.0)	1(100.0)
PROK2	1	0(0.0)	1(100.0)
PRAME	1	0(0.0)	1(100.0)
SNAI2	1	0(0.0)	1(100.0)
CD46	1	0(0.0)	1(100.0)
TRIM28	1	0(0.0)	1(100.0)
RBPJ	1	0(0.0)	1(100.0)
FCGR2B	1	0(0.0)	1(100.0)
IL9	1	0(0.0)	1(100.0)
GATA3	1	0(0.0)	1(100.0)
GDNF	1	0(0.0)	1(100.0)
GATA6	1	0(0.0)	1(100.0)
ACSL4	1	0(0.0)	1(100.0)
VIM	1	0(0.0)	1(100.0)
ZBTB16	1	0(0.0)	1(100.0)
FGF8	1	0(0.0)	1(100.0)
THRB	1	0(0.0)	1(100.0)
PRTN3	1	0(0.0)	1(100.0)
EML4-ALK	1	0(0.0)	1(100.0)
GRK2	1	0(0.0)	1(100.0)
NRP2	1	0(0.0)	1(100.0)
THBS1	1	0(0.0)	1(100.0)
TGFB2	1	0(0.0)	1(100.0)
WBP2	1	0(0.0)	1(100.0)
AGER	1	0(0.0)	1(100.0)
TNFAIP3	1	0(0.0)	1(100.0)
AICAR	1	0(0.0)	1(100.0)
ESR2	1	0(0.0)	1(100.0)
TSC22D3	1	0(0.0)	1(100.0)
RASSF3	1	0(0.0)	1(100.0)

F7	1	0(0.0)	1(100.0)
RASSF6	1	0(0.0)	1(100.0)
CLCA2	1	0(0.0)	1(100.0)
ING1	1	0(0.0)	1(100.0)
TFRC	1	0(0.0)	1(100.0)
NOTCH2	1	0(0.0)	1(100.0)
CSNK2B	1	0(0.0)	1(100.0)
TGFBR2	1	0(0.0)	1(100.0)
MTOR	1	0(0.0)	1(100.0)
CXCR4	1	0(0.0)	1(100.0)
ITGB1	1	0(0.0)	1(100.0)
WNT5A	1	0(0.0)	1(100.0)
FSH	1	0(0.0)	1(100.0)
TRAF6	1	0(0.0)	1(100.0)
SMAD3	1	0(0.0)	1(100.0)
RHOA	1	0(0.0)	1(100.0)
CD14	1	0(0.0)	1(100.0)
GDF2	1	0(0.0)	1(100.0)
NOD2	1	0(0.0)	1(100.0)
MAPK3	1	0(0.0)	1(100.0)
NFKBIB	1	0(0.0)	1(100.0)
MUC1	1	0(0.0)	1(100.0)
JUNB	1	0(0.0)	1(100.0)
SMARCB1	1	0(0.0)	1(100.0)
PLA2G10	1	0(0.0)	1(100.0)
CDK8	1	0(0.0)	1(100.0)
KLF6	1	0(0.0)	1(100.0)
IKBKG	1	0(0.0)	1(100.0)
CDK9	1	0(0.0)	1(100.0)
F10	1	0(0.0)	1(100.0)
FASLG	1	0(0.0)	1(100.0)
IRF6	1	0(0.0)	1(100.0)
SYK/ZAP	1	0(0.0)	1(100.0)
ITGA5	1	0(0.0)	1(100.0)
ACVRL1	1	0(0.0)	1(100.0)
PIM1	1	0(0.0)	1(100.0)
FIRRE	1	0(0.0)	1(100.0)
PDGFB	1	0(0.0)	1(100.0)
TSC22D1	1	0(0.0)	1(100.0)
TIRAP	1	0(0.0)	1(100.0)
C3	1	0(0.0)	1(100.0)
ST6GAL1	1	0(0.0)	1(100.0)
PPARA	1	0(0.0)	1(100.0)
NFKBIE	1	0(0.0)	1(100.0)
FCGR2A	1	0(0.0)	1(100.0)
AIMP1	1	0(0.0)	1(100.0)
LIF	1	0(0.0)	1(100.0)
BTC	1	0(0.0)	1(100.0)
PLAT	1	0(0.0)	1(100.0)
AVP	1	0(0.0)	1(100.0)
USF1	1	0(0.0)	1(100.0)
IFNL3	1	0(0.0)	1(100.0)
TICAM2	1	0(0.0)	1(100.0)
RTKN	1	0(0.0)	1(100.0)
CRH	1	0(0.0)	1(100.0)
ERVW-1	1	0(0.0)	1(100.0)
VCP	1	0(0.0)	1(100.0)
TSH	1	0(0.0)	1(100.0)
E2F3	1	0(0.0)	1(100.0)
TGFBR1	1	0(0.0)	1(100.0)
OGT	1	0(0.0)	1(100.0)
PRDM5	1	0(0.0)	1(100.0)
ESR1	1	0(0.0)	1(100.0)
KRAS	1	0(0.0)	1(100.0)
YY1	1	0(0.0)	1(100.0)
SIRT1	1	0(0.0)	1(100.0)
SMAD6	1	0(0.0)	1(100.0)
SKP2	1	0(0.0)	1(100.0)
CLU	1	0(0.0)	1(100.0)
FANCA	1	0(0.0)	1(100.0)
CBR3-AS1	1	0(0.0)	1(100.0)
SOX4	1	0(0.0)	1(100.0)
OPRM1	1	0(0.0)	1(100.0)
GAS6	1	0(0.0)	1(100.0)
ANGPT1	1	0(0.0)	1(100.0)
SNAI1	1	0(0.0)	1(100.0)
NPPA	1	0(0.0)	1(100.0)
APOA1	1	0(0.0)	1(100.0)
MTDH	1	0(0.0)	1(100.0)
HDL	1	0(0.0)	1(100.0)
PIK3CA	1	0(0.0)	1(100.0)
SGK1	1	0(0.0)	1(100.0)
PPARGC1A	1	0(0.0)	1(100.0)

ETV6	1	0(0.0)	1(100.0)
LPL	1	0(0.0)	1(100.0)
SOX11	1	0(0.0)	1(100.0)

[a] Number of MPM cell lines in which the specific upstream regulator was modulated ($p<0.05$ and Z-score ≥2 or Z-score ≤-2);

[b] number of MPM cell lines in which the specific upstream regulator was activated ($p<0.05$ and Z-score ≥2);

[c] percentage of activation calculated as the ratio between the activation and modulation scores;

[d] number of MPM cell lines in which the specific upstream regulator was inhibited;

[e] percentage of inhibition calculated as the ratio between the inhibition and modulation scores.
