# Supplementary material for: Epigenetic Immune Remodeling of Mesothelioma Cells: A New Strategy to Improve the Efficacy of Immunotherapy
Source: Epigenomes. 2021 Dec 14;5(4):27. doi: 10.3390/epigenomes5040027 (PMC8715476; doi:10.3390/epigenomes5040027)
Supplement: Supplementary file 1 [file epigenomes-05-00027-s001.zip › Table S5.pdf]

Supplemental Table S5. Gene specific expression nCounter data in MPM cell lines treated with guadecitabine vs untreated ones

	SARCOMATOID cells				BIPHASIC cells				EPITHELIOID cells	
	Meso3	Meso2	Meso11	Meso8	Meso4	Meso13	Meso5	Meso7	Meso1	Meso6
**CTA (#9)**										
MAGEA1	0.59 [a]	0.52	4.84	5.86	6.49	1.18	2.64	0.12	3.82	3.82
MAGEA12	0.89	2.86	-0.11	3.18	3.48	1.56	4.2	0.17	5.33	0.71
MAGEA3/A6	0.11	1.7	1.41	4.56	6.22	3.69	6.45	-0.06	-0.45	-0.2
MAGEA4	7.56	6.18	3.79	5.72	5.9	4.26	5.12	0.58	6.61	5.69
MAGEB2	2.99	7.43	4.24	2.67	6.87	6.45	6.39	0.54	6.98	7.67
MAGEC1	2.01	3.77	-0.11	0	2.32	0.32	-0.04	0.58	4.19	2.1
MAGEC2	0.17	6.59	-0.11	6.76	6.01	5.54	4.2	-3.12	6.97	6.74
CTAG1B	8.77	5.16	-0.11	2.44	7.59	3.95	4.79	6.57	6.78	5.9
CEP55	-0.17	0.16	0.19	0.88	0.7	0.01	1.3	0.05	4.78	0.8
	SARCOMATOID cells				BIPHASIC cells				EPITHELIOID cells	
**Type I_II_III IFN and IFN-responsive genes (#29)**	Meso3	Meso2	Meso11	Meso8	Meso4	Meso13	Meso5	Meso7	Meso1	Meso6
IFI16	-0.56	0.81	0.1	-0.26	-0.1	-0.07	0.37	-0.03	-0.42	0.83
IFI27	2.97	1.74	0.51	0.4	4.23	3.06	0	0.16	0.43	-0.21
IFI35	1.29	1.2	-0.07	0.79	-0.26	-0.21	0.28	-0.45	0.19	0.09
IFI6	2.75	2.67	-0.1	0.73	0.78	0.37	0.91	0.03	0.9	0.51
IFIH1	1.32	1.36	0.42	0.42	0.29	0.29	0.5	-0.4	-0.04	0.65
IFIT1	1.87	2.11	0.11	1.41	0.38	0.25	-1.21	-0.3	-0.14	-0.5
IFIT2	0.62	0.4	0.39	1.41	1.3	0.43	-0.57	-0.4	-1.38	-0.76
IFIT3	1.82	1.85	0.41	1.81	0.46	-0.16	-0.23	-0.23	-0.41	-0.49
IFITM1	2.36	2.25	0.37	1.9	2.88	1.77	0.87	0.26	0.34	-0.64
IFITM2	-1.21	-0.11	0.03	0.71	1.6	0.17	0.32	0.12	-0.7	-0.46
IFNA1	0.37	0.18	3.5	-2.73	-2.44	1.72	-0.4	-0.05	-2.66	3.11
IFNAR1	-0.27	-0.3	0.01	0.08	0.08	-0.42	-0.44	-0.48	-0.51	-0.27
IFNG	-0.76	1.39	-0.11	0	0	-2.06	-0.04	0.58	0.69	-0.25
IFNGR1	-0.44	-0.13	0.48	0.03	-0.11	0.3	-0.44	0.07	0.41	-0.25
IFNGR2	-0.52	-0.46	0.33	0.06	0.43	0.11	-0.64	0.1	-0.06	-0.98
OAS1	1.7	2.39	0.1	1.39	0	0.41	-0.02	-0.41	-0.59	0.78
OAS2	1.73	2.93	0.17	4.77	0	0.07	-0.43	-0.3	0.31	-0.16
OAS3	2.89	1.57	0.11	0.75	0.85	0.23	0.7	-0.15	-0.24	0.3
OASL	3.59	0.03	-0.02	2.18	4.69	-1.24	-1.41	0.07	0.05	1.14
IRF1	0.28	0.63	0.11	-0.02	0.03	-0.49	-0.16	-0.18	-0.14	-0.33
IRF2	-0.35	0.15	0.19	-0.25	-0.16	-0.42	-0.38	0.08	-0.1	0.58
IRF3	0.34	0.81	0.03	0.28	0.47	1.16	0.5	-0.31	-0.31	0.79
IRF4	0.03	-2.01	2.07	0	0	-0.14	-1.04	-0.62	1.41	-0.25
IRF5	-2.45	-0.11	2.45	-0.4	0.02	-0.32	-0.42	0.98	-0.68	2.09
IRF7	1.45	1.52	-0.63	0.95	1.57	3.19	1.3	-0.98	-0.32	1.09
IRF8	0.03	2.16	-0.11	0.71	0	0.32	1.85	0.62	0.06	-0.25
IRF9	0.84	0.95	0.01	-0.23	0.05	-0.25	-0.38	-0.4	0.62	1.09
ISG15	3.67	2.33	0.13	2.28	1.68	0.54	0.45	0.06	-0.49	-0.04
MX1	6.64	1.91	0.16	1.4	0.77	-0.16	0.14	-0.85	1.16	1.3
	SARCOMATOID cells				BIPHASIC cells				EPITHELIOID cells	
	Meso3	Meso2	Meso11	Meso8	Meso4	Meso13	Meso5	Meso7	Meso1	Meso6
**HLA class I (#5)**										
HLA-A	0.21	1.01	-0.04	0	0.18	-0.23	0.01	0.06	-0.34	-0.09
HLA-B	-0.22	0.61	-0.15	-0.32	-0.12	-0.15	-0.11	0.08	-0.51	0.01
HLA-C	-0.8	-1.18	0.38	0.4	0.82	0.4	-0.69	-1.51	1.12	-0.73
TAP1	1.28	1.56	0.31	0.56	0.31	-0.43	-0.46	0.05	0.53	0.2
TAP2	0.47	0.39	-0.16	-0.3	0.15	-0.3	-0.15	-0.32	-0.53	-0.4
	SARCOMATOID cells				BIPHASIC cells				EPITHELIOID cells	
**Positive and negative costimulation (#14)**	Meso3	Meso2	Meso11	Meso8	Meso4	Meso13	Meso5	Meso7	Meso1	Meso6
CTLA4	4.75	2.66	-0.11	1.87	3.83	3.55	2.36	0.87	0.06	-0.25
LAG3	1.62	0.96	2.23	0.51	3.48	1.45	-1.56	0.61	-0.64	-0.17
ICOS	0.03	0	-0.11	0	0	0.32	-0.04	0.58	0.06	-0.25
ICOSLG	-2.04	0.62	0.24	-0.63	-0.4	0.74	-1.38	-0.78	-0.19	1.02
CD28	0.03	0	-0.11	-0.79	0	0.32	-0.04	0.58	0.06	-0.25
CD40	4.14	2.74	2.18	0	0	0.69	3.93	-0.03	3.31	-0.88
CD40LG	0.03	0	-0.11	0	0	0.32	-0.04	0.58	0.06	-0.25
TIGIT	0.03	2.66	-0.11	0	0	0.32	-0.04	0.58	1.41	2.33
CD274	-0.25	0.32	0.21	0.71	2.16	4.44	2.64	-0.22	-1.12	-0.23
PDCD1	0.03	2.66	-0.11	2.18	0.92	2.07	-0.04	0.58	0.06	-1.02
PDCD1LG2	-0.7	0.61	-0.19	0.54	0.78	0.28	1.64	0.08	-0.34	-0.31

CD86	0.03	0	-0.11	0	0	0.32	-0.04	0.58	0.06	1.09
PVR	-0.66	0.34	-0.39	-0.86	0.35	0.14	0.29	0.13	-0.16	-0.12
CD276	-0.36	-0.83	-0.25	0.06	0.12	-0.14	-0.25	0.25	-0.44	-0.46

	**SARCOMATOID cells**				**BIPHASIC cells**				**EPITHELIOID cells**	
**Cytokines (#43)**	Meso3	Meso2	Meso11	Meso8	Meso4	Meso13	Meso5	Meso7	Meso1	Meso6
IL10	-2.72	-1.3	-0.11	0	0	-1.12	-0.04	0.58	0.06	1.51
IL10RA	0.03	0	-0.11	-0.79	0	0.32	-1.55	0.58	0.06	3.33
IL11	1.72	2.99	0.28	2.42	1.9	-0.39	1.53	0.18	-0.24	0.78
IL11RA	-0.12	-0.37	0.97	-0.16	0.22	0.6	1.24	-0.09	0.22	0.08
IL12RB2	2.2	2.85	-0.11	0	0	0.32	3.05	0.58	0.06	-0.25
IL15	-0.24	0.66	1.26	0.4	1.87	1.25	-0.55	-0.32	0.77	0.74
IL16	0.03	0	-0.11	0	0	0.32	-0.04	2.66	0.06	-0.25
IL17A	0.03	-0.52	-0.11	-1.41	-2.28	0.32	-3	0.58	0.06	0.5
IL18	-0.66	0.31	0.64	-0.62	0.74	-0.49	-0.26	0.37	0.58	0.04
IL18R1	-2.31	-0.03	-0.66	-0.93	-1.71	0.05	-1.41	-0.38	-0.77	-0.06
IL1A	1.66	1.22	-0.49	-0.1	0.52	1.18	1.84	-0.4	0.74	1.03
IL1B	0.21	0.86	-1.23	-0.54	0	-0.11	-0.04	-0.21	0.42	-0.64
IL1R2	3.76	-2.01	-0.11	-0.09	0	4.16	-3.6	1.73	0.88	3.19
IL1RN	0.03	0	-0.11	0.34	0	-1.5	-0.04	-1.77	-0.14	0.84
IL2	-2.21	0	-0.11	0	0	0.32	-0.04	-0.52	1.41	2.1
IL21R	0.03	0	-0.11	0	2.56	0.32	-0.04	0.58	0.06	1.84
IL22RA1	1.25	-0.44	3.33	0.03	2.19	-0.21	1.01	0.03	1	2.2
IL24	0.01	2.85	0.07	3.56	1.38	-1.5	2.69	0.92	4.62	3.33
IL2RA	0.03	0	-0.11	0	2.32	0.32	-0.04	0.58	0.06	2.69
IL2RB	3.25	0	-0.11	0	0	0.32	4.32	-0.06	0.06	1.67
IL2RG	4.37	5.68	-0.11	4.67	0.92	3.55	5.7	0.58	0.06	3.22
IL32	-0.46	1.81	-0.29	-0.27	0.83	0.92	0.07	-0.19	-0.07	2.06
IL33	-2.45	0	-0.27	-0.82	0	0.32	-0.04	0.86	-1.79	-0.25
IL34	-2.74	0.91	0.7	3.86	0.11	-0.77	-0.97	-2.03	0.69	1.22
IL4	0.03	-0.74	-0.11	-1.84	0	0.32	-1.52	0.58	0.06	-0.25
IL6	1.66	1.32	0.01	-0.32	-1.09	3.23	0.84	-0.5	0.82	3.79
IL6R	3.07	1.93	2.18	0.11	2.74	0.38	3.36	0.58	2.33	3.15
IL7R	-1.68	1.29	1.2	1.14	1.55	1.8	0.99	0.17	0.06	4.81
CSF1	-0.75	-0.18	0.45	-0.65	-1.02	-0.02	-2.37	0.08	3.31	0.19
CSF1R	-0.13	0.84	2.18	4.11	-0.34	-0.6	3.41	2.83	2.07	1.22
CSF2	1.22	4.6	0.12	3.32	0	-0.6	0.33	2.42	0.51	0.99
CSF2RB	0.03	0	-0.11	0	0	0.32	-0.04	0.58	0.06	-0.25
CSF3	0.03	0	3.04	0	0	0.32	-0.04	-0.18	0.06	-0.25
CSF3R	0.03	-0.87	1.41	0	1.42	-1.12	-0.04	1.86	0.06	-0.25
LIF	-0.1	-0.05	-0.49	-0.43	0	0.15	-1.14	-0.2	-0.71	-0.2
JAK1	-0.87	-0.11	0.04	-0.26	-0.11	0.08	-0.1	-0.04	-0.32	-0.66
JAK2	-0.14	0.12	0.63	0.49	-0.02	0.4	0.26	0.02	0.47	-0.46
JAK3	0.03	0	0.8	0	0	0.32	1.49	0.68	3.92	-0.25
STAT1	1.45	0.48	0.2	0.5	0.03	0.24	0.07	-0.21	0.25	0.9
STAT2	-0.09	0.7	0.13	-0.34	0.02	-0.24	-0.13	0.04	-0.27	0.02
STAT3	0	0.16	0.65	0.29	0.16	-0.1	-0.03	0.15	0.07	-0.19
STAT4	1.39	0.19	1.09	0	1.41	0.32	3.6	-1.16	1.46	-0.06
SPP1	1.68	5.52	-0.11	2.91	0	0.32	-0.04	0.44	0.06	-0.25

	**SARCOMATOID cells**				**BIPHASIC cells**				**EPITHELIOID cells**	
**Chemokines and receptors (#35)**	Meso3	Meso2	Meso11	Meso8	Meso4	Meso13	Meso5	Meso7	Meso1	Meso6
CX3CL1	1.89	1.86	1.22	0	3.91	0.32	3.42	2.27	1.41	4.69
CX3CR1	0.03	0	-0.11	0	0	-0.63	-0.04	0.58	0.06	-0.25
CXCL1	3.17	2.91	0.51	-0.28	3.38	0.4	-0.83	-0.15	0.32	0.31
CXCL10	-0.76	0.69	1.46	2.62	0	-0.86	-0.04	-0.69	2.98	3.84
CXCL11	1.81	1.02	0.51	3.95	2.08	0.43	-3.47	-0.21	5.63	-0.99
CXCL12	-1.87	0	-0.11	0	0	0.32	-0.04	0.58	0.06	1.09
CXCL13	0.03	0	-0.11	0	-1.11	0.32	-0.04	0.58	1.89	-0.25
CXCL14	0.84	-0.19	3.44	3.01	1.44	0.92	2.67	0.58	2.08	-0.44
CXCL16	1.9	0.62	1.45	0.8	0.14	0.14	0.51	-0.06	-0.44	0.23
CXCL2	1.07	1.31	0.35	-0.25	6.21	1.27	-0.33	-0.29	0.16	1.04
CXCL3	0.95	1.44	0.21	0.81	2.46	0.77	-0.8	-0.56	0.57	1.63
CXCL5	0.03	0	3.79	0	0	0.32	-0.04	-0.96	0.06	-0.25
CXCL6	0.03	0	4.28	0	0	2.07	1.85	0.11	2.21	0.27
CXCL8	2.68	3.33	0.14	0.19	4.65	0.32	0.29	0.14	0.69	0.38
CXCL9	0.03	-0.74	-0.11	0	0	0.32	-0.04	0.58	0.06	-0.25
CXCR2	2.68	1.09	-1.83	0	0	-2.06	-1.59	-1.46	2.25	1.58
CXCR3	0.03	0	-0.11	0	0	0.77	0.33	0.58	0.06	-0.25
CXCR4	3.84	0.84	4.41	3.32	1.53	0.2	0.87	2.27	0.06	-0.25
CXCR6	0.03	0	-0.49	0	0	0.32	-0.04	0.58	0.06	-0.25
CCR2	0.03	0.04	-0.11	0	0	0.32	-0.04	0.58	0.69	0.5
CCR4	6.84	4.12	3.58	1.34	4.3	-1.05	1.53	0.58	3.46	3.43

CCR5	-2.37	1.49	2.29	0	0	0.23	-2.8	-1.07	0.69	2.33
CCL13	0.03	-0.74	-0.11	0	0.16	0.32	-0.04	0.58	0.06	-0.25
CCL14	0.03	-0.74	-0.11	0	0	0.32	-0.04	0.58	0.06	-0.25
CCL18	-0.26	-0.13	0.13	-0.04	0.21	0.45	-0.44	0.76	-0.12	-0.73
CCL19	0.03	0	-0.11	-3.03	-1.11	0.32	-0.04	0.58	0.06	1.51
CCL2	-0.06	0.65	1.33	0.57	0.4	0.16	-0.94	-0.18	1.89	-0.25
CCL20	6.95	5.64	1.93	4.69	3.75	3.82	4.09	1.18	0.94	8.72
CCL21	0.03	-1.97	2.67	0	3.14	0.32	-0.04	0.58	-2.43	0.5
CCL22	0.03	0	1.84	0	0	0.32	-0.04	0.58	3.46	3.61
CCL3/L1	3.25	1.83	-0.11	0	0	0.32	-0.04	1.27	0.06	0.75
CCL4	0.03	0	-0.11	-0.79	-1.11	1.56	0.33	0.58	0.06	-0.25
CCL5	3.25	0.28	1.66	3.03	2.89	0.27	-0.41	0.52	0.06	3.11
CCL7	2.43	0.62	4.74	0	0	1.98	0.33	0.58	0.06	-0.25
CCL8	0.03	-2.27	2.87	0	2.53	0.92	-3.47	2.97	1.89	-0.25

	**SARCOMATOID cells**				**BIPHASIC cells**				**EPITHELIOID cells**	
**Regulation of Inflammation (#19)**	Meso3	Meso2	Meso11	Meso8	Meso4	Meso13	Meso5	Meso7	Meso1	Meso6
F2RL1	5.78	1.26	0.6	4.23	1.55	0.32	2.14	-0.42	-0.33	-0.05
PTGS2	0.18	0.01	3.33	0.54	2.44	0.71	-0.08	-0.09	-1.51	-0.75
NOD2	2.68	-2.49	2.18	0	1.78	-0.14	-0.25	0.56	3.92	1.54
ADORA2A	-0.06	-1.65	0.51	0.53	0.58	-0.37	-1.28	-1.32	0.04	0.82
RELB	0.32	0.82	-0.1	-0.37	0.22	0.08	-0.21	-0.44	-0.13	0.26
NLRP3	0.32	0	-0.11	1.43	0	0.32	-0.04	-0.1	-0.29	-0.39
APOE	-2.66	2.3	-0.11	1.43	3.71	1.03	2.5	0.11	0.06	-0.25
NFKB2	0.32	0.48	0.12	-0.16	0.27	0.01	-0.35	-0.19	-0.44	0.46
NFKBIA	-0.3	0.36	0.45	0.15	0.4	0	-0.63	0.08	0.2	-0.29
IKBKG	0.08	-0.07	0.14	0.24	-0.13	0.13	0.46	-0.18	0.48	0.45
A2M	-4.16	0	-0.49	0.26	0	0.32	0.33	0.58	3.31	2.1
RELA	-0.28	0.1	-0.28	-0.27	-0.08	0.24	-0.08	-0.05	-0.27	-0.41
RIPK2	-0.56	0.88	-0.13	-0.47	0.08	-0.37	-0.13	-0.48	0.12	0.41
CHUK	0.43	0.09	-0.05	0.18	0.06	0.01	0.32	-0.29	-0.13	-0.35
S100A8	0.03	0	-0.11	0	0	0.32	0.66	0.58	5.71	4.65
S100A12	0.03	2.43	0.8	5.18	0	1.56	-0.04	1.67	2.18	0.42
SBNO2	-0.46	0.08	-0.06	-0.77	0.07	-0.28	-0.88	-0.13	-0.33	-0.2
IKBKB	0.15	0.54	0.58	0.1	0.19	0.36	-0.76	-0.1	0.54	-0.37
NFKB1	-0.4	0.08	0.1	-0.55	0.07	0.07	-0.24	-0.64	-0.25	-0.29

[a] Values represent gene specific expression nCounter data (Log2 ratio)
