# Supplementary material for: Epigenetic Immune Remodeling of Mesothelioma Cells: A New Strategy to Improve the Efficacy of Immunotherapy
Source: Epigenomes. 2021 Dec 14;5(4):27. doi: 10.3390/epigenomes5040027 (PMC8715476; doi:10.3390/epigenomes5040027)
Supplement: Supplementary file 1 [file epigenomes-05-00027-s001.zip › Table S6.pdf]

**Supplemental Table S6. Real-Time PCR and nCounter expression values of selected genes in MPM cell lines treated with guadecitabine vs untreated ones**

| | NY-ESO-1 | | MAGE-A1 | | CDH1 | | CDH2 | | IFN-γ | | IFNGR | | IL-1β | | IL-6 | | IL-10 | |
|---|---|---|---|---|---|---|---|---|---|---|---|---|---|---|---|---|---|---|
| | nCounter | qRT-PCR | nCounter | qRT-PCR | nCounter | qRT-PCR | nCounter | qRT-PCR | nCounter | qRT-PCR | nCounter | qRT-PCR | nCounter | qRT-PCR | nCounter | qRT-PCR | nCounter | qRT-PCR |
| Meso3 Untreated | 1.1[a] | 8.13E-05[b] | 681.14 | 4.45E-02 | 11.78 | 2.10E-04 | 2843.92 | 6.05E-02 | 1.91 | 3.83E-07 | 636.15 | 7.36E-03 | 144.55 | 1.29E-03 | 111.64 | 4.08E-04 | 7.39 | 2.85E-05 |
| Meso3 Guadecitabine 1 µM | 479.75 | 5.30E-02 | 1022.06 | 1.36E-01 | 73.29 | 1.92E-03 | 1626.13 | 7.94E-02 | 1.12 | 2.07E-05 | 468.52 | 1.63E-02 | 167.61 | 3.07E-03 | 351.75 | 4.11E-03 | 1.12 | 1.26E-05 |
| Meso2 Untreated | 1 | 1.74E-05 | 196.64 | 1.53E-02 | 4.03 | 7.00E-05 | 4040.26 | 2.38E-01 | 1.00 | 7.67E-07 | 549.70 | 1.42E-02 | 133.95 | 3.56E-04 | 673.32 | 3.39E-03 | 2.46 | 1.11E-05 |
| Meso2 Guadecitabine 1 µM | 35.79 | 3.49E-03 | 281.70 | 3.03E-02 | 48.68 | 2.87E-03 | 2251.79 | 9.72E-02 | 2.63 | 1.20E-06 | 500.91 | 1.46E-02 | 243.94 | 1.69E-03 | 1676.14 | 1.07E-02 | 1.00 | 1.02E-04 |
| Meso11 Untreated | 1.45 | 1.26E-05 | 1.45 | 5.70E-06 | 12.06 | 8.48E-06 | 5633.46 | 1.31E-01 | 1.45 | 7.66E-07 | 1306.29 | 2.91E-02 | 2699.29 | 6.04E-02 | 17731.82 | 1.70E-01 | 1.45 | 1.45E-06 |
| Meso11 Guadecitabine 1 µM | 1.35 | 5.96E-04 | 41.61 | 3.25E-04 | 36.22 | 3.30E-04 | 4309.50 | 1.06E-01 | 1.00 | 3.55E-06 | 1823.72 | 3.72E-02 | 1153.74 | 2.08E-02 | 17819.53 | 1.29E-01 | 1.35 | 8.48E-06 |
| Meso8 Untreated | 1 | 9.80E-06 | 1.00 | 4.80E-06 | 9.10 | 6.24E-06 | 2166.34 | 5.84E-02 | 1.00 | 6.37E-07 | 890.06 | 1.88E-02 | 54.26 | 1.40E-03 | 4786.18 | 4.87E-02 | 1.00 | 5.10E-07 |
| Meso8 Guadecitabine 1 µM | 5.43 | 8.08E-04 | 58.22 | 1.19E-03 | 133.76 | 2.01E-03 | 1680.97 | 3.77E-02 | 1.00 | 1.83E-05 | 9.11 | 1.64E-02 | 37.29 | 9.24E-04 | 3822.48 | 2.44E-02 | 1.00 | 1.98E-05 |
| Meso4 Untreated | 1 | 2.18E-04 | 1.00 | 4.96E-05 | 3526.60 | 1.64E-01 | 732.23 | 6.22E-02 | 1.00 | 1.83E-06 | 611.75 | 3.67E-02 | 1.00 | 4.94E-04 | 1624.32 | 4.05E-02 | 1.00 | 7.95E-05 |
| Meso4 Guadecitabine 1 µM | 192.95 | 2.33E-02 | 90.08 | 3.51E-03 | 3864.86 | 1.04E-01 | 533.30 | 2.66E-02 | 1.00 | 4.79E-06 | 566.56 | 2.63E-02 | 1.00 | 1.73E-04 | 763.81 | 2.06E-02 | 1.00 | 3.80E-04 |
| Meso13 Untreated | 1 | 7.04E-06 | 11.78 | 5.29E-06 | 1089.07 | 1.62E-02 | 439.07 | 9.43E-03 | 1.00 | 1.70E-06 | 886.60 | 6.51E-03 | 1344.26 | 3.65E-04 | 10516.59 | 2.24E-04 | 1.00 | 0.00E+00 |
| Meso13 Guadecitabine 1 µM | 15.42 | 1.98E-03 | 26.63 | 5.58E-04 | 1206.74 | 1.87E-02 | 598.62 | 9.46E-03 | 1.50 | 4.93E-07 | 928.45 | 6.88E-03 | 1594.18 | 4.50E-04 | 7456.80 | 1.44E-03 | 1.50 | 5.92E-07 |
| Meso5 Untreated | 1.03 | 2.44E-04 | 9.04 | 7.90E-05 | 4658.08 | 2.22E-01 | 967.86 | 6.91E-02 | 5.19 | 7.73E-07 | 322.76 | 6.25E-02 | 23.33 | 2.09E-04 | 17.56 | 1.16E-02 | 2.71 | 7.00E-06 |
| Meso5 Guadecitabine 1 µM | 28.25 | 1.02E-02 | 56.54 | 5.56E-03 | 3275.11 | 1.92E-01 | 833.16 | 6.79E-02 | 1.25 | 1.03E-05 | 397.98 | 6.08E-02 | 21.65 | 1.23E-04 | 164.95 | 2.52E-02 | 1.25 | 1.53E-06 |
| Meso7 Untreated | 1 | 7.20E-05 | 34.80 | 2.77E-03 | 94.16 | 1.77E-03 | 978.61 | 3.28E-02 | 1.03 | 7.25E-07 | 1033.70 | 2.66E-02 | 1.00 | 6.00E-02 | 440.10 | 1.29E-01 | 1.03 | 9.87E-06 |
| Meso7 Guadecitabine 1 µM | 94.79 | 9.62E-03 | 37.81 | 3.13E-03 | 159.26 | 2.02E-03 | 857.92 | 1.76E-02 | 1.00 | 9.20E-07 | 764.30 | 1.79E-02 | 1.03 | 3.90E-02 | 789.92 | 6.17E-02 | 1.00 | 5.37E-06 |
| Meso1 Untreated | 1.04 | 1.14E-05 | 6.84 | 1.75E-05 | 11283.90 | 1.78E-01 | 1.04 | 3.30E-05 | 1.04 | 7.85E-06 | 542.76 | 7.67E-03 | 18.25 | 3.34E-04 | 159.22 | 1.63E-03 | 1.04 | 8.13E-06 |
| Meso1 Guadecitabine 1 µM | 113.92 | 8.10E-03 | 96.65 | 1.29E-03 | 7115.91 | 1.28E-01 | 1.08 | 1.88E-05 | 1.68 | 4.10E-06 | 721.52 | 1.26E-02 | 24.34 | 8.24E-04 | 281.20 | 2.86E-03 | 1.08 | 5.00E-06 |
| Meso6 Untreated | 1.19 | 2.10E-05 | 5.59 | 4.53E-05 | 5231.79 | 7.81E-02 | 1.19 | 2.22E-05 | 1.19 | 8.79E-07 | 1825.78 | 2.79E-02 | 40.09 | 6.65E-04 | 44.85 | 3.83E-04 | 1.19 | 1.54E-07 |
| Meso6 Guadecitabine 1 µM | 71.02 | 3.45E-03 | 78.72 | 6.31E-04 | 6696.13 | 1.11E-01 | 2.54 | 9.00E-05 | 1.00 | 4.49E-06 | 1534.71 | 2.52E-02 | 25.65 | 4.59E-04 | 620.54 | 5.26E-03 | 3.40 | 2.90E-07 |

[a] Values represent gene specific expression nCounter data (Log2 ratio)

[b] Data are reported as the number of immune molecules-specific mRNA normalized to the number of β-actin molecules, obtained by qRT-PCR analysis
